# Viral Tracing Confirms Paranigral Ventral Tegmental Area Dopaminergic Inputs to the Interpeduncular Nucleus Where Dopamine Release Encodes Motivated Exploration

**DOI:** 10.1523/ENEURO.0282-22.2022

**Published:** 2022-01-12

**Authors:** Susanna Molas, Rubing Zhao-Shea, Timothy G. Freels, Andrew R. Tapper

**Affiliations:** Department of Neurobiology, Brudnick Neuropsychiatric Research Institute, University of Massachusetts Chan Medical School, Worcester, Massachusetts 01605

**Keywords:** anxiety, dopamine, interpeduncular nucleus, motivation, novelty, ventral tegmental area

## Abstract

Midbrain dopaminergic (DAergic) neurons of the ventral tegmental area (VTA) are engaged by rewarding stimuli and encode reward prediction error to update goal-directed learning. However, recent data indicate that VTA DAergic neurons are functionally heterogeneous with emerging roles in aversive signaling, salience, and novelty, based in part on anatomic location and projection, highlighting a need to functionally characterize the repertoire of VTA DAergic efferents in motivated behavior. Previous work identifying a mesointerpeduncular circuit consisting of VTA DAergic neurons projecting to the interpeduncular nucleus (IPN), a midbrain area implicated in aversion, anxiety-like behavior, and familiarity, has recently come into question. To verify the existence of this circuit, we combined presynaptic targeted and retrograde viral tracing in the dopamine transporter-Cre mouse line. Consistent with previous reports, synaptic tracing revealed that axon terminals from the VTA innervate the caudal IPN; whereas, retrograde tracing revealed DAergic VTA neurons, predominantly in the paranigral region, project to the nucleus accumbens shell, as well as the IPN. To test whether functional DAergic neurotransmission exists in the IPN, we expressed the genetically encoded DA sensor, dLight 1.2, in the IPN of C57BL/6J mice and measured IPN DA signals *in vivo* during social and anxiety-like behavior using fiber photometry. We observed an increase in IPN DA signal during social investigation of a novel but not familiar conspecific and during exploration of the anxiogenic open arms of the elevated plus maze. Together, these data confirm VTA DAergic neuron projections to the IPN and implicate this circuit in encoding motivated exploration.

## Significance Statement

Ventral tegmental area (VTA) dopamine (DA) neurons respond to reward but can also be engaged by aversive stimuli highlighting the need to functionally characterize VTA projections to understand how DA signaling underlies motivated behavior. Previous studies identified VTA DA neurons that project to the interpeduncular nucleus (IPN) where they modulate anxiety and novelty preference. In mice, the existence of IPN-projecting VTA DA neurons was confirmed using viral tracing. Expressing a genetically encoded DA sensor in the IPN and monitoring DA revealed that IPN DA is increased in response to novel and anxiogenic stimuli. These data verify that a small population of DA neurons in the VTA project to the IPN where they are engaged during motivated exploration.

## Introduction

The modulatory neurotransmitter dopamine (DA) plays critical roles in reward, learning, motivation, and action selection (Schultz et al., 1997; Berridge and Robinson, 1998; Floresco, 2015; Arber and Costa, 2022). Despite decades of intense research, the precise regulation and the circuitry architecture of DAergic neurotransmission still remain unclear (Berke, 2018). Growing evidence demonstrate that midbrain DAergic systems are integrated by a spectrum of molecularly, anatomically, and functionally distinct neuron subtypes. In addition, single-cell gene expression profiling (Tiklová et al., 2019; Poulin et al., 2020; Phillips et al., 2022), together with projection-specific functional mapping, support the hypothesis that heterogeneous DA neuronal clusters can influence individual behavioral readouts (Lammel et al., 2014; Morales and Margolis, 2017; Poulin et al., 2018).

Midbrain DA neurons in the ventral tegmental area (VTA) respond to reward (Mirenowicz and Schultz, 1996), reward-predictive cues (Flagel et al., 2011), associative learning (Saunders et al., 2018), as well as salient stimuli, such as novel social investigations (Gunaydin et al., 2014; Solié et al., 2022). In addition, some VTA DA neurons are engaged by aversive stimuli (Matsumoto and Hikosaka, 2009) or during anxiety-related and fear-related behaviors (Zweifel et al., 2011). Most VTA DA neurons send abundant projection-specific outputs to the ventral striatum nucleus accumbens (NAc) region, where they regulate reward-related and aversive processing (Lammel et al., 2012; de Jong et al., 2019), encode saliency (Kutlu et al., 2021), or promote social behaviors (Gunaydin et al., 2014), but whether the same neurons send functional projections to additional areas and how they control emotional and motivational behaviors are not fully understood.

Medial and ventral to the VTA resides the interpeduncular nucleus (IPN) of the midbrain. The IPN receives excitatory inputs from the epithalamic medial habenula (mHb) and sends efferent projections to midbrain and hindbrain structures including the raphe, tegmentum, and pontine nucleus (Groenewegen et al., 1986; Lima et al., 2017). IPN neurons are predominantly GABAergic, although IPN glutamatergic and serotonergic neurons have also been reported (Quina et al., 2017; Sherafat et al., 2020). Anatomically, the IPN has been subdivided into the following three unpaired and four paired subnuclei: the median, unpaired subnuclei include the apical nucleus (IPA), rostral nucleus (IPR), and central nucleus (IPC), whereas the paired subnuclei include the dorsolateral (IPDL), dorsomedial (IPDM), lateral (IPL), and intermediate (IPI) subnuclei (Hemmendinger and Moore, 1984). The cytoarchitecture, molecular profiling, and functional connectivity of distinct IPN neuronal clusters is largely unknown.

Increasing attention has focused on the mHb–IPN axis over the last 2 decades, as it highly expresses a unique combination of nicotinic acetylcholine receptor (nAChR) subunits, α5, α3, and β4, encoded within the *CHRNA5-A3-B4* gene cluster (Improgo et al., 2010), extensively associated with nicotine dependence in human genetic studies (Berrettini et al., 2008; Bierut et al., 2008). Numerous investigations in rodents have corroborated the role of the mHb–IPN circuit as key regulator of nicotine intake (Fowler et al., 2011; Frahm et al., 2011) and of nicotine withdrawal, including both physical and affective aspects (Salas et al., 2009; Görlich et al., 2013; Antolin-Fontes et al., 2015; Zhao-Shea et al., 2013, 2015; Casserly et al., 2020; Klenowski et al., 2022). Emerging evidence further implicates this axis in regulating fear-related memories as well as baseline anxiety-like behaviors (Yamaguchi et al., 2013; Soria-Gómez et al., 2015; Zhang et al., 2016a; Molas et al., 2017a; Seigneur et al., 2018).

Recent data described a mesointerpeduncular pathway consisting of VTA DAergic neurons that innervate the IPN (Zhao-Shea et al., 2015), a circuit that mediates anxiety-like behavior through unique IPN microcircuitry (DeGroot et al., 2020) and that controls the motivational component of familiar social investigations (Molas et al., 2017b). Such cross talk between two adjacent midbrain structures with apparent opposing roles in regulating behavior (Wills et al., 2022) could have important implications for balancing motivational and affective behaviors. However, a recent study excluded the existence of an anatomic connection from the VTA to the IPN (Nasirova et al., 2021). Thus, a comprehensive analysis clarifying VTA DAergic neuron connections to the IPN and elucidating internal signals that trigger DA release in this brain area, would provide valuable insight into VTA DA neuron architecture, as well as intrinsic midbrain DA circuitry function.

## Materials and Methods

### Animals

All animal experiments were conducted in accordance with the *Guide for the Care and Use of Laboratory Animals* provided by the National Research Council, and with approved animal protocols from the Institutional Animal Care and Use Committee of the Institution. C57BL/6J [stock #000664, The Jackson Laboratory (https://www.jax.org/strain/000664)] and DAT-Cre [stock #006660, The Jackson Laboratory (https://www.jax.org/strain/006660)] mice were bred in the institution animal facility. Cre lines were crossed with C57BL/6J mice, and only heterozygous animals were used for the experiments. Mice of both sexes were used in all experiments. For social experiments, juvenile stimuli always consisted of C57BL/6J mice (4–7 weeks old). Mice were group housed with a maximum of five per cage and were kept on a standard 12 h light/dark cycle (lights on at 7:00 A.M.) with *ad libitum* access to food and water. Following viral brain injections and recovery overnight, mice for behavior experiments were transferred to the reverse light/dark cycle room (lights on at 7:00 P.M.) for 3 weeks before fiber brain implantations or additional experiments. Mice were single housed for at least 1 week before behavior testing, which was conducted during the dark cycle (8:00 A.M. to 5:00 P.M.).

### Viral preparations

Biosensors, and optogenetic and control plasmids packaged into viral particles were purchased from Addgene. For tracing experiments, we used pAAV.hSyn.mCherry [2.6 × 10^13^ genome copies (GC)/ml; catalog #114472-AAV2 (https://www.addgene.org/114472/)], pAAV.hSyn.DIO.EGFP [1.4 × 10^13^ GC/ml; catalog #50457-AAVrg (https://www.addgene.org/50457/)], pAAV-hSyn-Flex-mGFP-2A-Synaptophysin-mRuby (7.0 × 10^11^ GC/ml; catalog #71760-AAV1 (https://www.addgene.org/71760/)], and pAAV-hSynapsin1-Flex-axon-GCaMP6s [2.2 × 10^13^ GC/ml; catalog #112010-AAV5 (https://www.addgene.org/112010/)]. For fiber photometry experiments, we used pAAV.hSyn.dLight1.2 [8.7 × 10^12^ GC/ml; catalog #111068-AAV5 (https://www.addgene.org/111068/)]. Viral injections were performed on 6-week-old mice, and mice were allowed to recover for 4-6 weeks to allow for transgene expression.

### Stereotaxic surgeries

Briefly, mice (6 weeks old) were deeply anesthetized with a mixture of 100 mg/kg ketamine and 10 mg/kg xylazine (VEDCO) by intraperitoneal injection. Ophthalmic ointment was applied to maintain eye lubrication. The skin of the skull was shaved and disinfected with iodine. Mice were then placed on a heating pad and in a stereotaxic frame (Stoelting), and the skull was exposed by making a small incision with a scalpel blade. Using bregma and λ as landmarks, the skull was leveled along the coronal and sagittal planes. A 0.4 mm drill was used for craniotomies at the target bregma coordinates. Microinjections were made by using a gas-tight 33 ga Hamilton 10 μl neurosyringe (catalog #1701RN, Hamilton) and a microsyringe pump (Stoelting). The following coordinates [in mm, from bregma) were used: for NAc: anteroposterior (AP), 1.0; mediolateral (ML), ±0.5; dorsoventral (DV), −4.0; for VTA: AP, −3.51; ML, ±0.2; DV, −4.2; and for IPN: AP, −3.51; ML, −1; DV, −4.81; and 12° angle. Viral volumes for injections were 300 nl, delivered at a constant flow rate of 30 nl/min. After injection, the needle was left unmoved for 10 min before being slowly retracted. The incision was then closed and held together with Vetbond.

After 3 weeks of recovery from virus injection, mice underwent surgery as described above for the implantation of optic fibers. Optic fiber [core diameter, 200 μm; numerical aperture (N.A.), 0.48; Doric Lenses] was placed targeting the IPN (AP, −3.8 mm; ML, −1 mm; DV, −4.61 mm; at 12.5°) and was held in place with adhesive luting cement (C&B Metabond, Parkell) followed by dental cement (Cerebond, PlasticsOne). Mice were allowed to recover for 5–7 d in the reverse light/dark cycle room before behavior tests. Injection sites and viral expression were confirmed for all animals by experimenters blinded to behavioral outcome, as previously described (Molas et al., 2017b). Animals showing no viral or off-target site viral expression or incorrect optic fiber placement (<10%) were excluded from analysis.

### Fiber photometry and data analysis

Fluorescent signals from biosensors were recorded with a Fiber Photometry System (Doric Instruments). An LED driver was used to deliver excitation light from LEDs at 465 nm (output, ∼8.5 mW) and at 405 nm (output, ∼5 mW), which was used as an isosbestic wavelength for the indicator (Doric Instruments). The light was reflected into a 200 μm, 0.48 N.A. optic fiber patch cord via the Dual Fluorescence Minicube (Doric Instruments). Emissions were detected with a femtowatt photoreceiver (model 2151, Newport) and were amplified by transimpedance amplification to give an output voltage readout. Sampling (12 kHz) and lock-in demodulation of the fluorescence signals were controlled by Doric Neuroscience Studio software with a decimation factor of 50. A Doric Instruments behavior camera was connected to the Doric Neuroscience Studio software using a USB 3.0 Vision interface to synchronize the photometry recordings with time-locked behavioral tracking systems. All mice were habituated to the patch cord plugged to the optic fiber implant for 10 min in their home cages before the start of the experiment. For social novelty tests, recordings began with the animal in the home cage for 1 min and then placed by the experimenter to the center of the behavioral apparatus. Behavioral events were tallied from the videos in a blinded fashion, and analysis was done using the time-locked photometry recording.

Fiber photometry data analysis was performed using custom-written MATLAB and Python scripts. A low-pass filter (3 Hz) was applied to the demodulated fluorescence signals before the 405 nm channel was scaled to the 465 nm by applying a least mean squares linear regression. Scaled signals were used to calculate the Δ*F*/*F*_0_, where Δ*F*/*F*_0_ = (465 nm signal – fitted 405 nm signal)/fitted 405 nm signal. The *z*-scores were calculated using as a baseline the average Δ*F*/*F*_0_ values from the −1.0 s before the onset of each behavioral event (considered as time 0, *t* = 0). For random sampling, two sets of 10 start time stamps were randomly generated, one set within the first 5–149.99 s and the other within 150–294.99 s of the 5 min recording trace. For the 20 random time stamps, Δ*F*/*F*_0_ were extracted from −1 to 3 s, and the *z* scores of each event were estimated using as baseline the −1.0 s before the time stamp.

### Behavioral assays

Animals were acclimated to the testing room for 30 min before any experimental assay, and all testing was performed under dim red-light conditions.

#### Social behavior

Social behavior experiments were performed in wild-type C57BL/6J mice expressing the dLight1.2 biosensor in the IPN. Both male and female mice were used, which interacted with a same-sex C57BL/6J juvenile conspecific. Animals were tested in a Plexiglas apparatus (42 × 64 × 30 cm) containing two plastic grid cylinders (diameter, 25 × 10 cm) located at opposite corners of a rectangular maze. Subject mice were first habituated to the apparatus and the empty cylinders for a 5 min period. Following habituation, a juvenile unfamiliar C57BL/6J conspecific (4–7 weeks of age) was placed inside one of the two cylinders (counterbalanced), reducing social investigations led by the subject animals. The subject mouse was then positioned in the central zone and allowed to freely explore the social and nonsocial cylinders for 5 min. This testing phase was repeated for 24 h, on day 2, using the same juvenile conspecific located in the same compartment, which became familiar. The apparatus and cylinders were cleaned with Micro-90 Solution (International Products Corporation) to eliminate olfactory traces after each session. All sessions were video recorded and synchronized to activity dynamics. Exploration of the social and nonsocial cylinders in videos of the trials were labeled frame by frame by experimenters blind to group conditions. The onset of each behavioral exploratory event (considered as *t* = 0) was defined whenever the subject mouse directed its nose toward the cylinders at a distance of <2 cm and initiated a sniffing investigation. Sitting or resting next to the cylinder or objects was not considered exploration.

#### Elevated plus maze

The elevated plus maze (EPM) apparatus consisted of a central junction (5 × 5 cm) and had four arms elevated 45 cm above the floor with each arm positioned at 90° relative to the adjacent arms. Two closed arms were enclosed by high walls (30 × 5 × 15 cm) and the open arms were exposed (30 × 5 × 0.25 cm). A 60 W red fluorescent light was positioned 100 cm above the maze and was used as the illumination source. Both male and female C57BL/6J mice expressing the DA biosensor dLight1.2 in the IPN were used. The optic fiber implant was connected to the recording patch cord, and then mice were placed on the junction part of the maze facing one of the open arms. All mice were given 5 min of free exploration while their behavior was video recorded and synchronized to the dLight1.2 signals via the Doric Instruments fiber photometry system, as described above.

### Immunostaining and microscopy

Mice were euthanized by injection of sodium pentobarbital (200 mg/kg, i.p.) and transcardially perfused with ice-cold 0.1 m PBS, pH 7.4, followed by 10 ml of cold 4% (w/v) paraformaldehyde (PFA) in 0.1 m PBS. Brains were postfixed in 4% PFA for 2 h and then submerged in 30% sucrose. Brains were sliced to coronal sections (25 μm) by using a freezing microtome (model HM430, Thermo Fisher Scientific). For virus expression and fiber implant verification, after washes in 0.1 m PBS, sections were mounted, air dried, and coverslipped with Vectashield Mounting Medium (Vector Laboratories). Slices were imaged using a fluorescence microscope (MicroImmagine, Carl Zeiss) connected to computer-associated image analyzer software (release 4.6.1, AxioVision). For immunohistochemical staining, brain sections were permeabilized with 0.2% Triton X-100 in 0.1 m PBS for 5 min, blocked with 2% BSA in 0.1 m PBS for 30 min, and then incubated overnight with the corresponding primary antibodies in 2% BSA at 4°C. The following primary antibodies were used: mouse anti-tyrosine hydroxylase [TH; 1:500; catalog #MAB318, Millipore (https://www.emdmillipore.com/US/en/product/Anti-Tyrosine-Hydroxylase-Antibody-clone-LNC1,MM_NF-MAB318)]; and guinea pig anti-synaptophysin [1:300; catalog #AGP-144, Alomone Labs (https://www.alomone.com/p/guinea-pig-anti-synaptophysin-antibody/ANR-013-GP]. Slices were subsequently washed in 0.1 m PBS, blocked with 2% donkey (or goat) serum (Sigma-Aldrich) for 30 min and then incubated in secondary antibodies for 1 h [1:800; donkey anti-mouse 647 (catalog #A31571), goat anti-guinea pig 594 (catalog #A11076), Thermo Fisher Scientific]. After washes in 0.1 m PBS, sections were mounted, air dried, and coverslipped with Vectashield medium with DAPI (Vector Laboratories). Images were obtained using a confocal microscope (LSM 700, Zeiss) at 10× or at 10× with a 1.5 zoom. Images were analyzed using ImageJ Fiji to create a zoomed-in inset (the red line square on the images, with a 1.5 or 2 zoom factor). The ImageJ JAcoP method was used for colocalization analysis between VTA^DA^ → IPN fibers expressing AxonGCaMP and synaptophysin staining. Briefly, each image threshold was set automatically for analysis before Mander’s coefficient was applied to obtain the fraction of synaptophysin (red) overlapping with VTA^DA^ → IPN terminals (green) and vice versa. For quantification of fluorescently labeled axons from VTA^DA^ neurons innervating the IPN, the Digital Enhancement of Fibers with Noise Elimination (DEFiNE) method was applied (Powell et al., 2019; available for download at: https://figshare.com/s/1be5a1e77c4d4431769a). Axons were quantified in confocal images that were not processed through the clean images function, but each input image was a single-channel maximum intensity projection. Quantification was performed in ROIs (0.3 × 0.4 mm) randomly allocated within the anterior (bregma, −3.4 mm) and posterior (bregma, −3.8 mm) IPN.

### Statistical analysis

Statistical analyses for fiber photometry were performed using parametric tests on *z*-scored data after testing for normality. One-way or two-way repeated-measures (RM) ANOVA with Dunnett’s multiple-comparisons tests or Bonferroni’s *post hoc* tests was conducted for the analyses involving the comparison of group means, as indicated. The *z*-scores are presented as the mean ± SEM of all events for transitions between open arms (included junction) and closed arms, and for social approach behaviors. Comparisons of *z* scores were made using the calculated average for each animal. All analyses were performed using Prism 9 (GraphPad). Statistical significance was accepted at *p* < 0.05 (see Table 1 for statistics summary).

### Data availability

The code used for fiber photometry data analysis is freely available on GitHub (https://github.com/TapperLab/TapperLab) and also as Extended Data 1.

## Results

**Figure 1.**
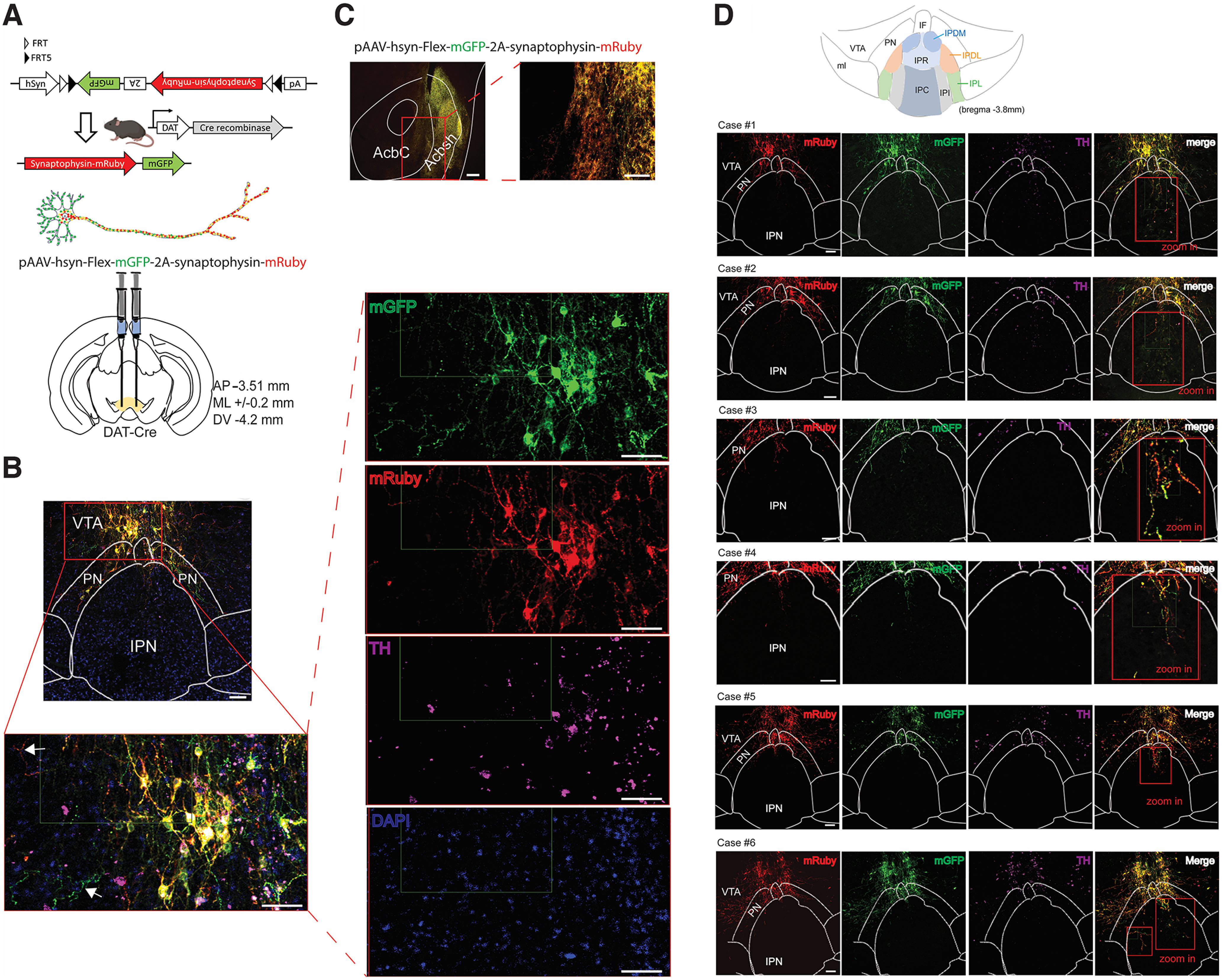
VTA DA neurons send axonal projections to the IPN. ***A***, Schematics depicting Cre-dependent recombination of the construct pAAV-hsyn-Flex-mGFP-2A-synaptophysin-mRuby in DAT-Cre mice and the viral injection strategy used. Dendritic arbors from a Cre^+^-transfected neuron display exclusive membrane-bound GFP (mGFP) fluorescence, whereas mRuby red fluorescence predominantly localizes in axon terminals. ***B***, Top, Representative image of viral injection in the VTA of DAT-Cre mice, showing mGFP (green) and mRuby (red) expression in DA neurons immunolabeled with TH staining (magenta). Nuclei are counterstained with DAPI (blue). Bottom, Magnified view of the inset region from the top image. White arrows show mGFP in dendritic arborizations and mRuby in axonal projections from VTA^DA^ transfected neurons. Scale bars, 100 μm. ***C***, Representative image showing mGFP and mRuby expression in efferents innervating the NAc from VTA^DA^ transfected neurons. Scale bars, 100 μm. ***D***, Illustrative drawing of the different IP subnuclei: IPA, IPC, IPDL, IPDM, IPI, IPL, and IPR. IF, Interfascicular nucleus; ml, medial lemniscus; PN, paranigral nucleus. All cases 1–6 (3 males, 3 females) show virally transfected neurons in the VTA colabeled with TH staining. Scale bars, 100 μm. Inset, Magnified views (red squares, 2× zoom in) demonstrate VTA^DA^ axon terminals (mRuby^+^) innervating the IPR and also the IPDM/IPDL regions.

We used a genetic strategy to target putative DA neuron subtypes and rigorously investigate DAergic projections from the VTA to the neighboring IPN. To this aim, we specifically selected a knock-in genetic mouse line that expresses Cre recombinase under the transcriptional control of the endogenous DA transporter (DAT) promoter. In this mouse line, Cre recombinase expression is driven from the 3′ untranslated region of the endogenous DAT gene by means of an internal ribosome entry sequence (IRES) to reduce interference with DAT function (Bäckman et al., 2006). Some neurons in the IPN express *Th* mRNA, which can lead to recombination in *Th*-IRES-Cre mice, although these neurons have low/undetectable TH protein in the adult brain (Poulin et al., 2018). These *Th*^+^ IPN neurons are not related to midbrain DA neurons, as they are not derived from the midbrain floor plane, and they lack the expression of typical DAergic neuronal markers such as DAT, NURR1, FOXA2, or PITX3 (Poulin et al., 2018); therefore, using DAT-Cre mice restricts and minimizes expression to midbrain DA neurons. Previous work expressed a Cre-dependent virus in the VTA of DAT-Cre animals and detected neuronal projections innervating mainly the caudal part of the IPN (cIPN; Molas et al., 2017b; DeGroot et al., 2020). To verify that VTA^DA^ → IPN projections are indeed axonal terminals and not simply DA dendritic elements extending into the IPN, we injected adeno-associated viruses (AAVs) containing the hSyn.Flex.mGFP.2A.synaptophysin.mRuby construct into the VTA of DAT-Cre mice (Fig. 1*A*). Following Cre recombination, synaptophysin fused to the mRuby red fluorophore is selectively transported into the axonal compartments of the transfected neurons (Fig. 1*A*; Zhang et al., 2016b). TH immunostaining demonstrated efficient recombination restricted to DA neurons in the midbrain (Fig. 1*B*). Furthermore, via circuit mapping, abundant axon terminals were detected in the NAc region, the principal output target of VTA^DA^ neurons (Fig. 1*C*). These VTA^DA^ → NAc axon terminals intensely expressed synaptophysin-mRuby fused protein (Fig. 1*C*), altogether validating the viral-mediated genetic strategy. To delineate the VTA^DA^ → IPN circuit, we used a group of six mice, with comparable results. All injected animals reliably exhibited VTA^DA^ synaptophysin–mRuby axon terminals innervating the IPR region of the cIPN (Fig. 1*D*). Additional VTA^DA^ axonal varicosities were also detected targeting the cIPN IPDM/IPDL subregion (Fig. 1*D*), consistent with previous data (Molas et al., 2017b; DeGroot et al., 2020).

**Figure 2.**
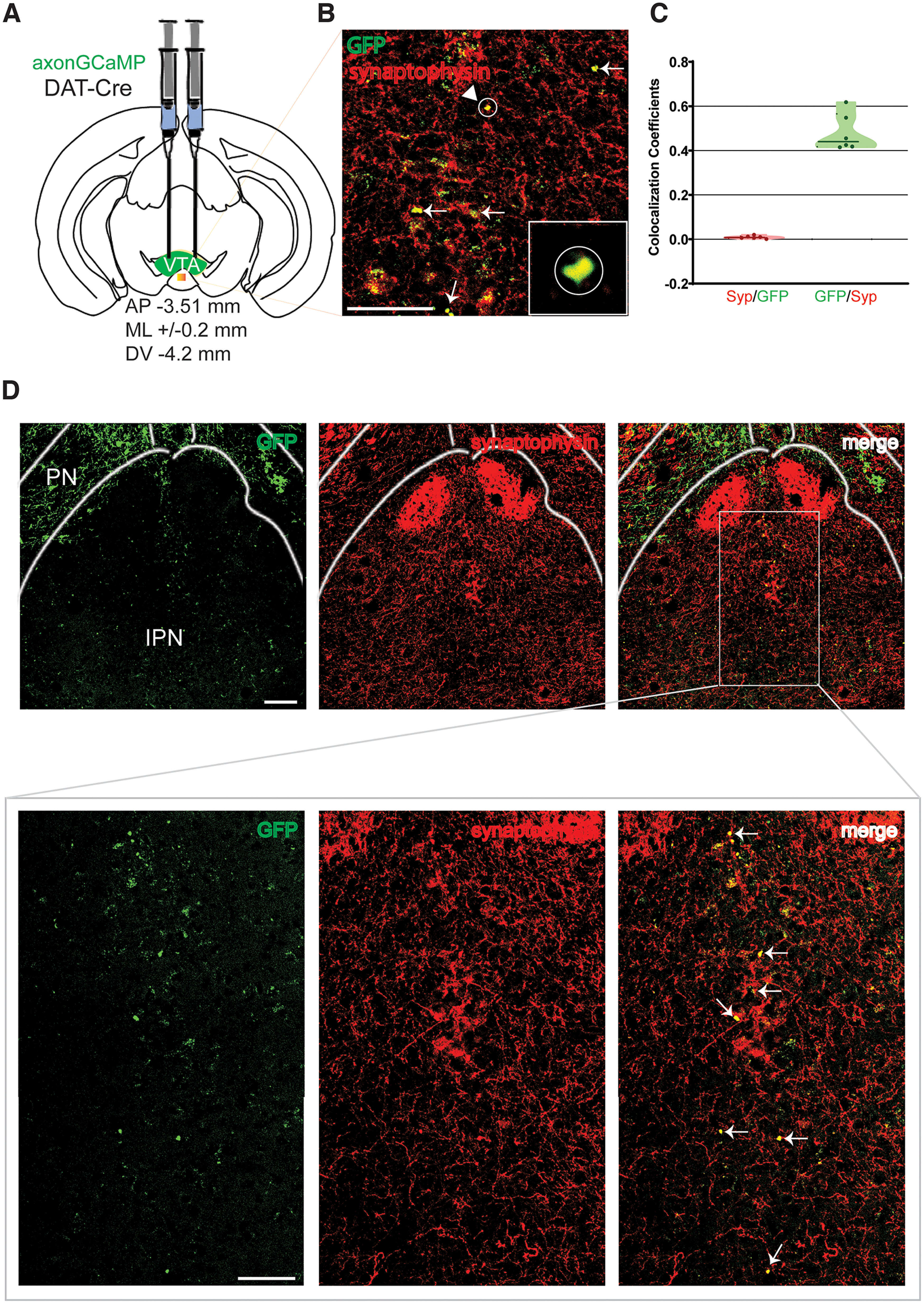
DAergic projections from the VTA to the IPN are presynaptic terminals. ***A***, Schematic of the viral injection strategy in the VTA of DAT-Cre mice. ***B***, Example of image showing eGFP (AxonGCaMP) colabeled with synaptophysin staining (red) in IPN. White arrows indicate presynaptic puncta colocalization. Inset, Magnified view of colocalization between eGFP and the synaptophysin marker. Scale bar, 100 μm. ***C*,** Quantification of the colocalization coefficient between eGFP and synaptophysin staining from single-plane confocal images containing the cIPN (*n* = 6 mice; 4 males, 2 females). ***D***, Top, AxonGCaMP expression in the VTA of DAT-Cre mice (eGFP, green), synaptophysin immunostaining (red) and colocalization of the two channels (merge) in brain slices containing the cIPN. Scale bar, 100 μm. Bottom, Enlarged view of the IPR region from the top images (gray square). White arrows denote VTA^DA^ eGFP^+^ presynaptic projections in the IPR colocalized with synaptophysin puncta. Scale bar, 100 μm.

To reassure that VTA DA neurons send neuronal projections innervating the neighboring IPN and that these are active presynaptic axons, DAT-Cre mice received an injection of Cre-dependent AxonGCaMP in the VTA expressed via AAV5-mediated gene delivery (Fig. 2*A*). This genetically encoded calcium indicator is uniformly enriched in axons, allowing for structure-specific labeling of presynaptic terminals (Broussard et al., 2018). Similarly, as described above, presynaptic terminals from VTA^DA^ neuronal inputs were observed in the IPR and IPDM regions of the cIPN (Fig. 2*B–D*). Moreover, immunostaining against synaptophysin protein revealed robust colocalization between the GFP^+^ (AxonGCaMP) and synaptophysin (Fig. 2*B–D*), confirming active presynaptic structures.

**Figure 3.**
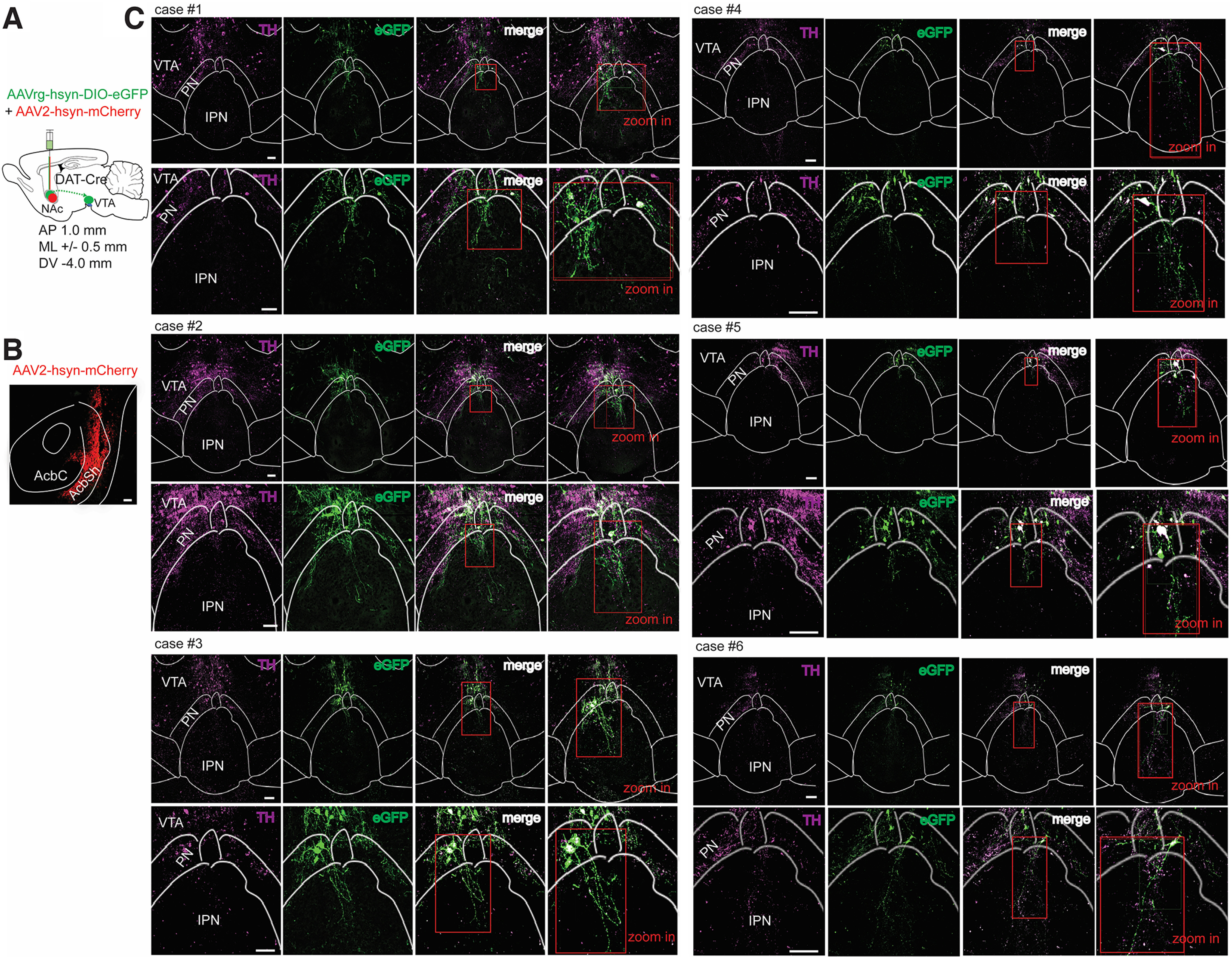
VTA^DA^ neurons from the PN send projections to the IPN. ***A***, Schematic of viral strategy used. DAT-Cre mice were injected with a viral mixture of AAV-hSyn-DIO-eGFP (retrograde) and AAV2-hSyn-mCherry (location marker; 1:1) into the NAc. ***B***, Representative image showing the virus injection site targeting the NAc shell area (AcbSh). Scale bar, 100 μm. ***C***, Example of injected animals, cases 1–6 (4 males, 2 females), all showing retrolabeled eGFP^+^ neurons in the VTA colabeled with TH staining. For each case: top, TH immunostaining (magenta), retrolabeled eGFP^+^ neurons from the NAc (green), and overlay of the two channels (merge) in brain slices containing the cIPN. Scale bar, 100 μm. Insets: right, a magnified view enclosing the PN and IPR in the merged channel (red square, 2× zoom in); bottom, enlarged view of the PN and IPR region from the top images with a right inset image of the merge channel demonstrating AcbSh-projecting neurons in the PN region are DAergic (TH^+^) and also send efferents to the IPR in the cIPN (red square, 2× zoom in). Scale bar, 100 μm.

**Figure 4.**
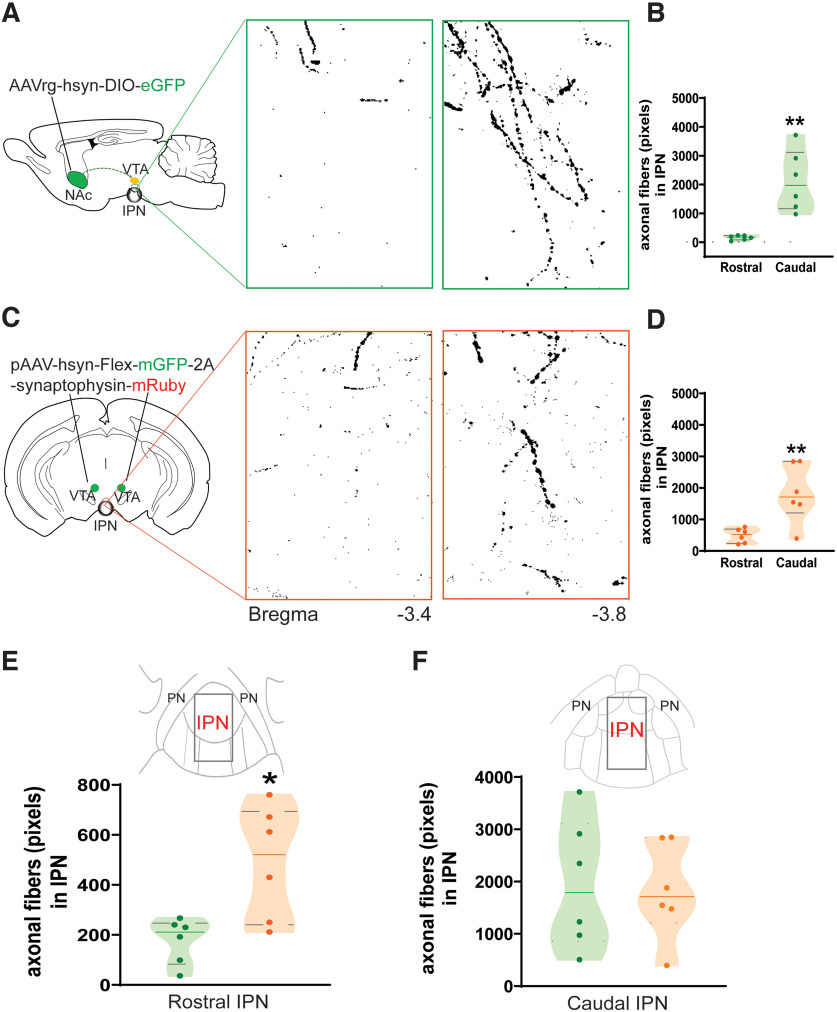
DEFiNE quantification of fluorescently labeled axons from VTA^DA^ neurons to the IPN. ***A***, Viral injection schematics (left) and representative images of axonal fibers innervating the IPN from retrolabeled eGFP^+^ AcbSh-projecting VTA^DA^ neurons after DEFiNE processing at anterior (−3.40 mm) and more posterior (−3.80 mm) IPN bregma (right). ***B***, DEFiNE quantification of the retrolabeled AcbSh-VTA^DA^ axonal fibers innervating the anterior and posterior IPN represented as total pixel count (*n* = 6 mice; unpaired two-tailed *t* test: *t*_(10)_ = 4.546, **p = 0.0011). ***C***, Schematic of the pAAV-hsyn-Flex-mGFP-2A-synaptophysin-mRuby viral strategy used in DAT-Cre mice for labeling VTA^DA^ neurons (left) with representative images of their axonal fibers innervating the IPN after DEFiNE processing at anterior (−3.40 mm) and more posterior (−3.80 mm) bregma (right). ***D***, DEFiNE quantification of the VTA^DA^ axonal fibers innervating the anterior and posterior IPN represented as the total pixel count (*n* = 6 mice; unpaired two-tailed *t* test: *t*_(10)_ = 3.438, **p = 0.0064). ***E***, Comparison of axonal fibers in the anterior IPN (bregma, −3.40 mm) quantified with the DEFiNE method when VTA DA neurons are directly transfected with the pAAV-hsyn-Flex-mGFP-2A-synaptophysin-mRuby construct versus retrolabeled eGFP^+^ AcbSh-projecting VTA^DA^ neurons (unpaired two-tailed *t* test: *t*_(10)_ = 3.114, *p = 0.011). ***F***, Same comparison as in ***E*** at IPN bregma −3.80 mm (unpaired two-tailed *t* test: *t*_(10)_ = 0.184, *p* = 0.8577).

Distinct VTA^DA^ projection populations regulate reward associations and motivation via specific NAc inputs (Heymann et al., 2020). To elucidate the projection specificity of VTA^DA^ that innervates the cIPN, DAT-Cre mice received a coinjection of AAV2-hsyn-mCherry (localization marker) together with AAVrg-hsyn-DIO-eGFP in the NAc region (Fig. 3*A*). Imaging of the target injection site confirmed viral-mediated gene delivery restricted mainly to the shell area of the NAc (Fig. 3*B*). In addition, to verify the retrolabeled neurons detected in the VTA were positive for DAergic markers, brain slices of the injected animals were immunostained against TH protein. All the experimental animals (*n* = 6 mice) exhibited abundant terminal projections from retrolabeled VTA → NAc projecting neurons that innervated the IPR region of the cIPN (Fig. 3*C*). The cell bodies from VTA → IPN projecting neurons mostly localized in the paranigral (PN) area of the VTA (Fig. 3*C*) and were indeed DAergic, as shown by colocalization with TH staining (Fig. 3*C*). For visualization enhancement and quantification of the fluorescently labeled axons, we used the DEFiNE method. Axonal fibers innervating the IPN from retrolabeled VTA^DA^ → NAc projecting neurons were highly enriched in posterior regions of the IPN (i.e., cIPN) compared with anterior IPN bregma (Fig. 4*A*,*B*). Similarly, quantification of axonal fibers originating from direct infusion of the synaptophysin–mRuby construct in VTA^DA^ neurons revealed increased axon terminals at more posterior IPN bregma compared with anterior (Fig. 4*C*,*D*). Noticeably, in anterior IPN bregma, the number of axonal fibers was higher when VTA^DA^ neurons were directly transfected with the synaptophysin–mRuby construct as opposed to retrolabeled VTA^DA^ → NAc projecting neurons (Fig. 4*E*). In contrast, these two viral-mediated VTA^DA^ neuron-labeling strategies resulted in a similar number of axonal fibers at posterior IPN bregma (Fig. 4*F*).

**Figure 5.**
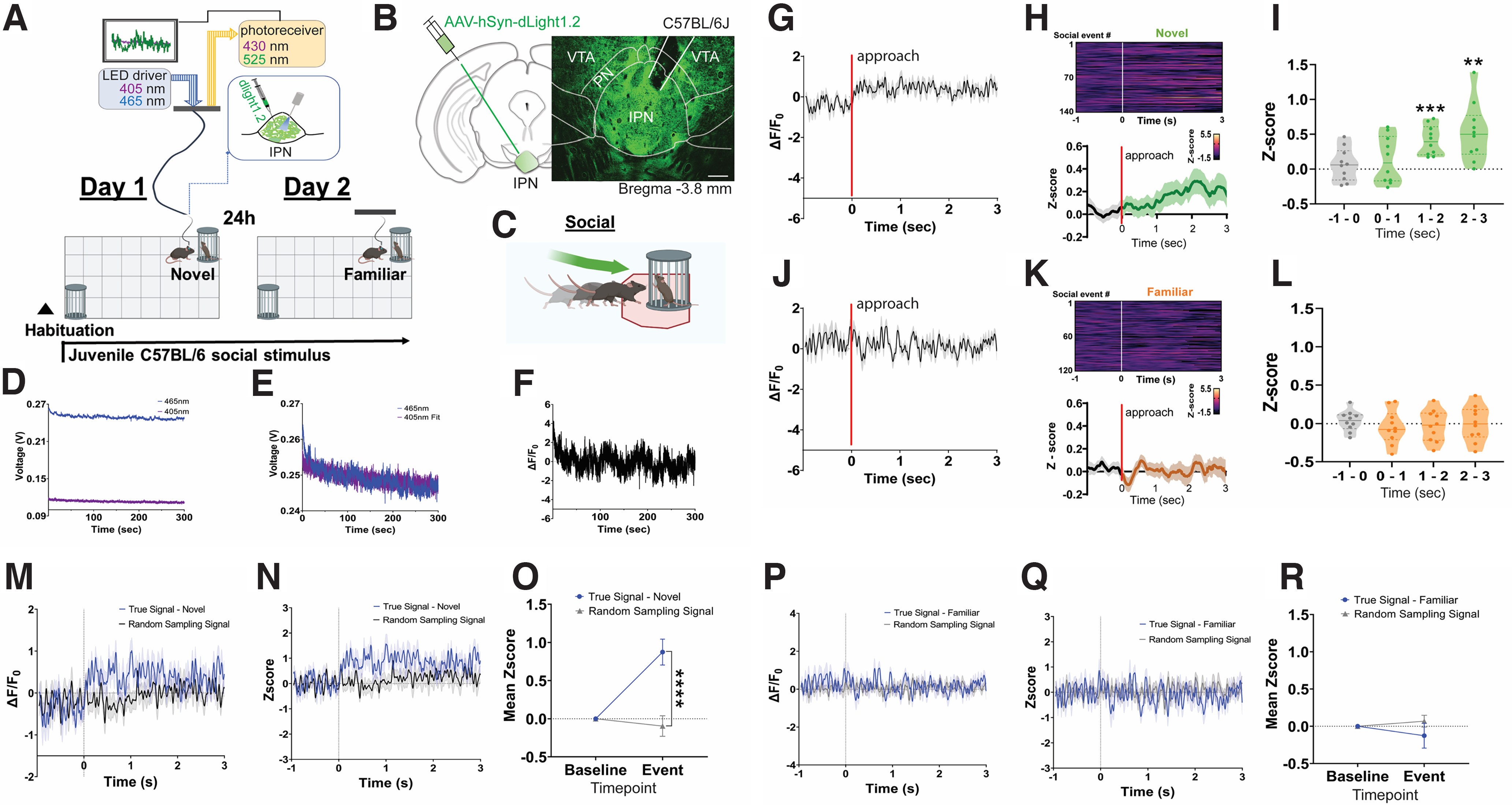
Novel social encounters trigger IPN DA signals. ***A***, Schematic of the experimental approach used to measure IPN DA activity during interactions with novel and familiar social stimuli. Subject mice were exposed to the same juvenile C57BL/6 conspecific on days 1 (novel) and 2 (familiar), while IPN DA signals were recorded using the dLight1.2 biosensor. ***B***, Schematic of AAV-dLight viral injection strategy in the IPN of C57BL/6 mice (left) and representative pictograph of DA sensor dLight1.2 (green) expression with optic probe location targeting the cIPN (right). Scale bar, 100 μm. ***C***, Illustration of a social sniffing investigation. ***D***, Example of raw signals (in volts) corresponding to the 465 and 405 nm channels recording during a 5 min interaction with a new social stimulus. ***E***, The 405 nm channel is scaled to the 465 nm by applying a least mean squares linear regression. ***F***, Scaled signals are used to calculate the Δ*F*/*F*_0_ value, where Δ*F*/*F*_0_ = (465 nm signal – fitted 405 nm signal)/fitted 405 nm signal. ***G***, Δ*F*/*F*_0_ values time locked to IPN DA signals relative to the initiation of a social sniffing investigation (red line) on day 1, when mice interact with a novel conspecific. ***H***, Heatmap representations (top) and *z*-score values (bottom) of the time-locked IPN DA signals relative to social novelty explorations. ***I***, Average *z* score per second compared with the baseline signal from 1 s before the onset of each social sniffing event (preonset, gray). Statistical comparisons were made using an average *z* score per animal (*n* = 10 mice; 6 males, 4 females). Significant increases in IPN DA activity were observed 2∼3 s after onset of novel social sniffing investigations. One-way RM ANOVA (*F*_(3,39)_ = 21.80, *p *<* *0.0001). Dunnett’s multiple-comparisons test: **p <* *0.01, ***p <* *0.001. ***J***, Δ*F*/*F*_0_ values time locked to IPN DA signals relative to the time initiating a social sniffing investigation (red line) on day 2, when mice interact with a familiar conspecific. ***K***, Heatmap representations (top) and *z*-score values (bottom) of time-locked IPN DA signals relative to familiar social explorations. ***L***, Average *z* score per second compared with the 1 s baseline signal demonstrate no significant change during familiar social sniffing investigations. One-way RM ANOVA (*F*_(3,39)_ = 0.7103, *p *=* *0.517). ***M***, Example of IPN DA Δ*F*/*F*_0_ values time locked to novel social investigations compared with Δ*F*/*F*_0_ values obtained with random sampling across the 5 min recording session. ***N***, The *z*-score values of ***M***. ***O***, Mean *z*-score values of the baseline and the 3 s novel social investigation event for the true signal compared with random sampling signal. Two-way RM ANOVA, significant time × *z*-score interaction; *F*_(1,29)_ = 19.13, *p* = 0.0001, Bonferroni’s *post hoc* test; ****p < 0.0001. ***P***, Example of IPN DA Δ*F*/*F*_0_ values time locked to familiar social investigations compared with Δ*F*/*F*_0_ values obtained with random sampling across the 5 min recording session. ***Q***, The *z*-score values of ***P***. ***R***, Mean *z*-score values of the baseline and the 3 s familiar social investigation event for the true signal compared with the random-sampling signal. All data represent the mean ± SEM.

Previous work suggested that the VTA^DA^ → IPN circuit is engaged during anxiety-like behaviors (DeGroot et al., 2020) and when mice encounter unfamiliar conspecifics (Molas et al., 2017b). Although DA signals have been detected in acute mouse IPN slices (DeGroot et al., 2020), the real-time dynamics of *in vivo* IPN DAergic neurotransmission have never been reported. To this aim, here we recorded IPN DA dynamics in freely behaving mice using the genetically encoded DA sensor dLight1.2 (Patriarchi et al., 2018). Fluctuations in IPN DA signals were recorded during the three-chamber sociability task, when mice encountered a new juvenile conspecific (Fig. 5*A*; Materials and Methods). On the following day, subject mice were presented to the same juvenile conspecific in the same location, which became familiar (Fig. 5*A*; Materials and Methods). To this aim, we virally expressed dLight1.2 in the IPN of C57BL/6J mice, enabling ultrafast optical DA recordings, and at 3 weeks post-viral transduction we implanted an optic fiber targeting the injection site (Fig. 5*B*). IPN DA dynamics were time locked to when animals approached and initiated a sniffing investigation of conspecific stimuli (Fig. 5*C*). Demodulated fluorescence signals were obtained from the 465 and 405 nm channels in a 5 min trial (Fig. 5*D*). The 405 nm channel was scaled to the 465 nm by applying a least mean squares linear regression (Fig. 5*E*). Scaled signals were used to calculate the Δ*F*/*F*_0_ where Δ*F*/*F*_0_ = (465 nm signal – fitted 405 nm signal)/fitted 405 nm signal (Fig. 5*F*). On day 1 of the sociability test, sniffing investigation of a novel conspecific significantly increased the release of DA in the IPN (Fig. 5*G–I*). However, IPN DA signals rapidly habituated on the next session, as the conspecific became familiar (Fig. 5*J–L*). Random sampling of IPN DA signals without being time locked to social sniffing investigations did not result in apparent changes in activity either when mice interacted with a novel conspecific (Fig. 5*M–O*) or when this became familiar (Fig. 5*P–R*).

**Figure 6.**
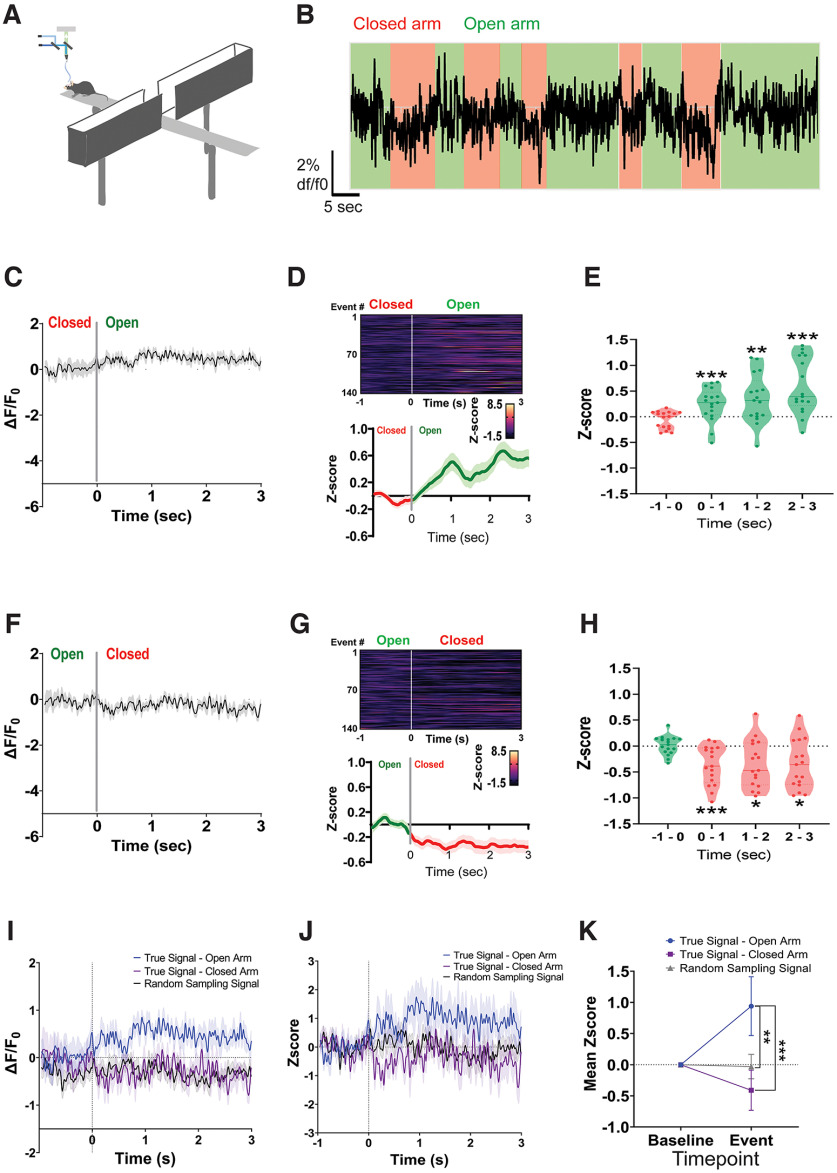
IPN DA signals are engaged with exploration of anxiogenic environments. ***A***, Schematic depicting fiber photometry recordings of IPN DA signals using the dLight1.2 biosensor in the EPM test. ***B***, Representative trace of IPN dLight1.2. fluorescence signals (d*F*/*F*_0_) when mice explored the open arms (green) versus the closed arms (red) of the EPM. ***C***, Δ*F*/*F*_0_ values time locked to IPN DA signals relative to the transition from the closed to the open arms of the EPM. ***D***, Heatmap representations (top) and *z*-score values (bottom) of time-locked IPN DA signals relative to the transition from the closed to open arms of the EPM (gray line). ***E***, Average *z* score per second compared with the baseline signal from 1 s before the exploration of the open arms. Statistical comparisons were made using an average *z* score per animal (*n* = 17 mice; 9 males, 8 females) that was calculated from all events. Significant increase in IPN DA activity was observed 1∼3 s postonset of open arm investigations. One-way RM ANOVA (*F*_(3,67)_ = 18.15, *p < *0.0001). Dunnett’s multiple-comparisons test: **p <* *0.01, ***p <* *0.001. ***F***, Δ*F*/*F*_0_ values time locked to IPN DA signals relative to the transition from the open to the closed arms of the EPM. ***G***, Heatmap representations (top) and *z*-score values (bottom) of time-locked IPN DA signals relative to the transition from the open to the closed arms of the EPM (gray line). ***H***, Average *z* score per second compared with the baseline signal from 1 s before the exploration of the closed arms. Significant decrease in IPN DA activity was observed 1∼3s after the onset of closed arm investigations. One-way RM ANOVA (*F*_(3,67)_ = 7.617, *p *=* *0.0042). Dunnett’s multiple-comparisons test: *p <* *0.05, ***p <* *0.001. ***I***, Example of IPN DA Δ*F*/*F*_0_ values time locked to the transition to the open or closed arms of the EPM compared with Δ*F*/*F*_0_ values obtained with random sampling across the 5 min recording session. ***J***, The *z*-score values of ***I***. ***K***, Mean *z*-score values of the baseline and the 3 s open and closed EPM arm exploratory event for the true signal compared with random sampling signal. Two-way RM ANOVA, significant time × *z* score interaction: *F*_(2,36)_ = 4.14, *p* = 0.024; *p* = 0.0001, Bonferroni’s *post hoc* test: **p < 0.001, ***p < 0.001. All data represent the mean ± SEM.

**Figure 7.**
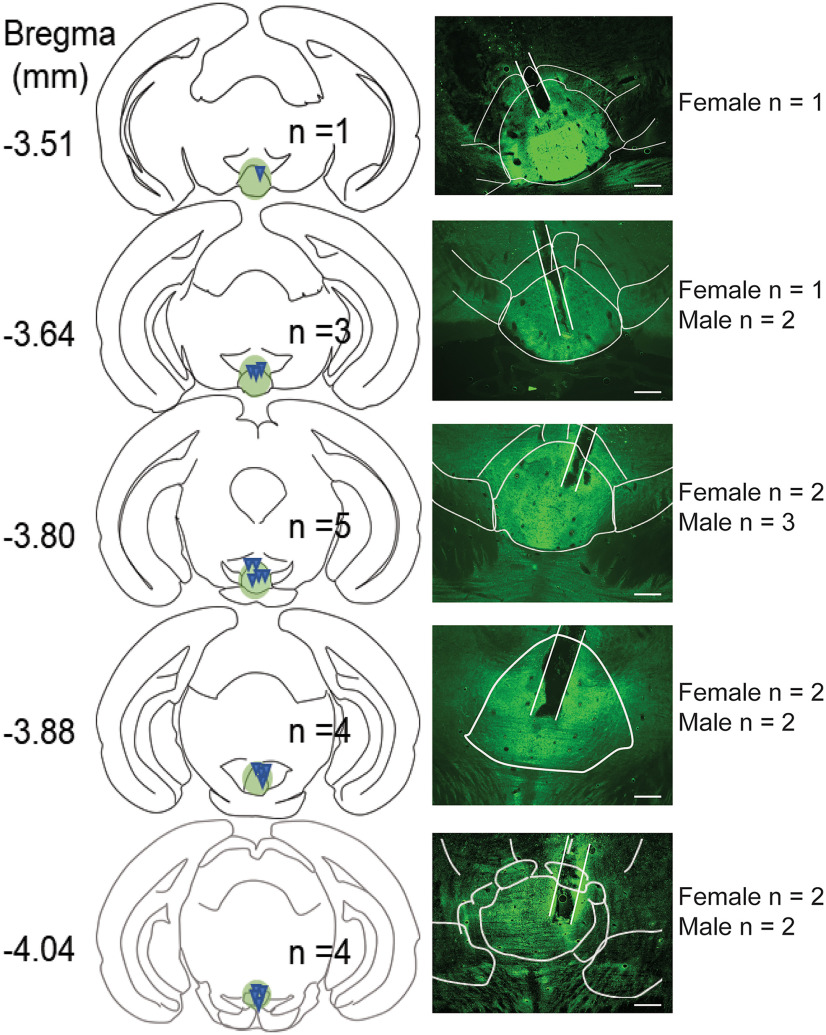
Distribution of fiber placement within the cIPN. Schematics and representative images of dLight1.2 biosensor expression in the IPN of C57BL/6 mice with examples of fiber placements distributed along the cIPN (bregma, −3.51 to −4.04 mm). Scale bar, 100 μm.

To further investigate IPN DA signals triggered by additional behaviors, we recorded IPN DA dynamics in mice tested in the EPM (Fig. 6*A*), a well established paradigm to measure anxiety-like behaviors in rodents (Walf and Frye, 2007). As mice investigated the open arms of the EPM, the release of DA in the IPN significantly increased (Fig. 6*B–E*). Conversely, the transition from the open to the closed EPM compartments led to reductions in IPN DA signals (Fig. 6*B*,*F–H*). Time-locked IPN DA signals when mice entered the open arms were higher compared with when entering the closed arms of the EPM or with non-time-locked random sampling signals (Fig. 6*I–K*). All the recorded animals were verified for correct viral expression and fiber placement within the cIPN (Fig. 7).

## Discussion

DA dysfunction has been implicated in numerous brain diseases, including addiction, depression, schizophrenia, Parkinson’s disease, and anxiety disorders (Horga and Abi-Dargham, 2019; Nestler and Lüscher, 2019; Ruitenberg et al., 2021; Taylor et al., 2022; Zalachoras et al., 2022). A comprehensive understanding of the circuit architecture and the functional mapping of DA neurons is imperative to gain insights into inherent regulation of DA neurotransmission in health and disease. The present study confirms the existence of a mesointerpeduncular pathway that connects the VTA with the IPN, thereby modulating behavioral states with implications in overall midbrain DA circuitry function.

Viral-mediated circuit tracing replicated previous findings (Zhao-Shea et al., 2015; Molas et al., 2017b; DeGroot et al., 2020), validating anatomic connections between VTA DAergic neurons and the IPN. The current work used the DAT-Cre knock-in mouse line, in which Cre mimics the expression pattern of the plasma membrane dopamine transporter (Bäckman et al., 2006; Lammel et al., 2015) and, therefore, demonstrates higher specificity targeting putative midbrain DA neurons (Poulin et al., 2018). VTA DA axons preferentially innervated the IPR region of the cIPN, as previously reported. These axon terminals were detected in most injected animals across multiple experimental cohorts and appeared to be more obvious in those mice where viral expression extended to the PN region of the VTA. In addition, synaptically targeted markers localized in terminal projections from the VTA^DA^ → IPN circuit, whereby protein immunostaining revealed active presynaptic terminals rather than passing fibers. Interestingly, the cIPN is highly enriched in neurons expressing the D_1_ receptor (Molas et al., 2017b), but also in serotonergic cell bodies (Groenewegen et al., 1986). Serotonergic IPN neurons innervate the ventral hippocampus (vHipp) to mediate active stress coping and natural reward (Sherafat et al., 2020). Considering that cIPN neurons can amplify VTA^DA^ signals through a microcircuit that spans to additional IPN subregions (DeGroot et al., 2020), if some of these cIPN neurons comprise the serotonergic IPN → vHipp pathway, then the VTA^DA^ signal would amplify to more distant regions to control motivational and affective behaviors.

Anatomical and functional connectivity of midbrain DA neurons has been broadly investigated across animal species (Swanson, 1982; Morales and Margolis, 2017). Numerous studies identified the source of synaptic input to DA neurons (Lammel et al., 2012; Watabe-Uchida et al., 2012; Beier et al., 2015), as well as output targets (Lammel et al., 2011, 2008; Poulin et al., 2018; Heymann et al., 2020). While consistent data indicate that the NAc is the major target of VTA DA neurons, additional structures such as the amygdala, cortex, hippocampus, ventral pallidum, septum, periaqueductal gray, bed nucleus of stria terminalis, olfactory tubercle, and locus coeruleus, among others, also receive DAergic inputs from the VTA. Noticeably, most of the circuit-tracing studies traditionally focus on those regions with the highest abundance of DA terminal projections, neglecting target-specific sites that receive sparse DAergic inputs. For instance, VTA neurons send local, topographically organized axonal connections that innervate the VTA itself (Adell and Artigas, 2004; Ferreira et al., 2008; Aransay et al., 2015), which overall have received less attention. Of note, Aransay et al. (2015) also reported VTA innervation to the IPN, which, although less frequent, nevertheless supports a direct anatomic link between the VTA and IPN.

The anatomic location of DA neuron synaptic output can be a critical factor determining its intrinsic properties and behavioral outcomes (Lammel et al., 2011; de Jong et al., 2019). Our data show that a subpopulation of NAc shell-projecting VTA DA neurons in the PN region may preferentially project into the IPN to innervate cIPN, as reported previously (DeGroot et al., 2020). Emerging evidence suggests that subpopulations of VTA DAergic neurons can innervate more than one brain structure (Aransay et al., 2015). Specifically, medial shell NAc-projecting DA neurons send significant collaterals outside the striatum, including the septum and ventral pallidum, indicating that this DA subpopulation is capable of simultaneously influencing neural activity in multiple brain regions (Beier et al., 2015). Since the same DA neurons presumably innervate the IPN, the data together position the IPN as an integral member within specific VTA DAergic subcircuitries.

Our photometry results demonstrate that innate DA signals in the IPN are triggered with motivated exploration, when mice investigate novel conspecific individuals and when they explore the anxiogenic arms of the EPM. These results affirm that social interactions bear rewarding aspects and recruit neural circuits of motivation (Chevallier et al., 2012), including DAergic systems (Gunaydin et al., 2014; Hung et al., 2017; Bariselli et al., 2018; Solié et al., 2022). Given that the NAc shell represents a storage site for social memories (Okuyama et al., 2016), one possibility could be that innate IPN signals contribute to social novelty and familiarity responses, supporting previous findings (Molas et al., 2017b). On the other hand, NAc shell-projecting VTA DA neurons are recruited by aversive stimuli and cues that predict them (de Jong et al., 2019). Increased IPN DA signals with the exploration of anxiogenic environments would result from activation of a neural network that strengthens responses to aversive stimuli to modulate anxiety-like behavior.

A recent study excluded the existence of an anatomic connection from the VTA to the IPN (Nasirova et al., 2021). One possible explanation for the discrepancy in the results may be that most of the viral-mediated circuit tracing in the study by Nasirova et al. (2021) was done in a Cre mouse line that only targets IPN neurons expressing the α5 nAChR subunit. Although neurons in the IPN are highly enriched in α5*-nAChRs (Ables et al., 2017), some subpopulations do not express the α5-encoding gene. Thus, limiting IPN circuit tracing to an α5-expressing neuronal subtype does not accurately reflect total IPN connectivity. In addition, for the viral-mediated retrograde tracing analysis, the authors selected IPN brain slices with a maximum IPN caudal bregma coordinate of −3.6 mm according to the atlas of Paxinos and Franklin (2001; Nasirova et al., 2021). As mentioned above, VTA DA neurons that project to the IPN localize more caudal, at coordinates −3.63 to −4.03 mm from bregma, which were likely missed in the analysis. Noticeably, previous work using rabies tracing from overall IPN neurons did detect sparse cell bodies localized in caudal VTA (Lima et al., 2017). Nasirova et al. (2021) used the Allen Connectivity Atlas to reinforce their negative data. However, the few Allen examples performed in the Slc6a3-Cre (DAT-Cre) line lack viral expression transfecting caudal VTA PN neurons, thereby precluding the detection of any putative VTA^DA^ innervation to the IPN. Additionally, Nasirova et al. (2021) included examples of VTA Cre-mediated anterograde tracing in DAT-Cre mice, but, for this experiment, the authors used a nonvalidated Cre-dependent synaptically targeted GFP marker, which presented strong labeling of cell bodies in the medial mamillary nucleus and also the IPN itself (Nasirova et al., 2021), two brain regions lacking DA neurons, thus raising questions regarding the specificity of the virus and therefore the validity of the results. Surprisingly, the article by Nasirova et al. (2021) failed to cite, consider, or discuss the study by DeGroot et al. (2020), which used a multidisciplinary approach and specifically demonstrated the following: (1) DA detection in IPN slices using a genetically encoded DA sensor; (2) optogenetic activation of VTA DA IPN inputs elicits a postsynaptic response that is blocked by a D_1_ receptor antagonist; (3) retrograde Cre-dependent AAV-eGFP injection into the medial nucleus accumbens shell labels VTA neurons that clearly project into the IPN of DAT-Cre mice (a result that was repeated here with the addition of TH staining to label DAergic neurons); and (4) optogenetic activation or silencing the DAergic IPN input decreases and increases anxiety-like behavior, respectively.

In summary, the present study was able to confirm the existence of a mesointerpeduncular pathway that connects the VTA with the IPN, replicating previous findings (Aransay et al., 2015; Zhao-Shea et al., 2015; Molas et al., 2017b; DeGroot et al., 2020). These results may significantly influence the prevailing models of intrinsic midbrain DA circuitry as well as of IPN function. Considering that VTA DAergic neurons also send projections to the mHb (Phillipson and Pycock, 1982; Beier et al., 2015), the data together suggest a complex direct dopaminergic modulation of the habenulointerpeduncular tract that may have strong impact on reward-related, aversive/affective motivated behaviors. Finally, beyond the VTA–IPN axis, and bearing in mind that the activation of small subsets of neuronal ensembles can lead to selective widespread activation of neural networks with concomitant behavioral outcome (Marshel et al., 2019; Dalgleish et al., 2020), the present work emphasizes the need of investigating sparse, functionally relevant neglected circuits that may serve as signal amplification to computationally process motivational information.

**Table 1:** Statistics summary for Figures 4–6

Figure	Data structure	Type of test	Value	Significance	95% confidence interval
4*B*	Normal distribution	Unpaired two-tailed *t* test	*t*_(10)_ = 4.546	*p* = 0.0011	1005–2937
4*D*	Normal distribution	Unpaired two-tailed *t* test	*t*_(10)_ = 3.438	*p* = 0.0064	472.4–2213
4*E*	Normal distribution	Unpaired two-tailed *t* test	*t*_(10)_ = 3.114	*p* = 0.011	88.7–535
4*F*	Normal distribution	Unpaired two-tailed *t* test	*t*_(10)_ = 0.184	*p* = 0.8577	−1530 to 1297
5*I*	Normal distribution	One-way RM ANOVA	*F*_(3,39)_ = 21.8	*p* < 0.0001	
		Dunnett's multiple comparisons test		−1 to 0 s vs 1–2 s; *p* < 0.001	−0.467 to −0.225
				−1 to 0 s vs 2–3 s; *p* < 0.01	−0.7073 to −0.2226
5*L*	Normal distribution	One-way RM ANOVA	*F*_(3,39)_ = 0.7103	*p* = 0.517	
5*O*	Normal distribution	Two-way RM ANOVA	Interaction *F*_(1,29)_ = 19.13	*p* = 0.0001	−0.6159 to −0.1622
		Bonferroni’s multiple-comparisons test		Event signal vs random, *p* < 0.0001	
5*R*	Normal distribution	Two-way RM ANOVA	Interaction *F*_(1,26)_ = 1.423	*p* = 0.2436	
6*E*	Normal distribution	One-way RM ANOVA	*F*_(3,67)_ = 18.15	*p* < 0.0001	
		Dunnett’s multiple-comparisons test		−1 to 0 vs 0–1; *p* = 0.0007	−0.4559 to −0.1324
				−1 to 0 vs 1–2; *p* = 0.0042	−0.6817 to −0.1301
				−1 to 0 vs 2–3; *p* = 0.0001	−0.958 to −0.3466
6*H*	Normal distribution	One-way RM ANOVA	*F*_(3,67)_ = 7.617	*p* = 0.0042	
		Dunnett’s multiple comparisons test		−1 to 0 vs 0–1; *p* = 0.0005	0.1783–0.5891
				−1 to 0 vs 1–2; *p* = 0.0152	0.0677–0.6580
				−1 to 0 vs 2–3; *p* = 0.032	0.029–0.6929
6*K*	Normal distribution	Two-way RM ANOVA	Interaction *F*_(2,36)_ = 4.136	*p* = 0.0242	−0.54 to 0.2074
		Bonferroni’s multiple-comparisons test		Signal open vs close, *p* = 0.0008	
				Signal open vs random, *p* = 0.0047	

10.1523/ENEURO.0282-22.2022.ext1 Extended Data 1 The code used for fiber photometry data analysis. Download Extended Data 1, ZIP file.

## Synthesis

Reviewing Editor: Christie Fowler, University of California, Irvine Decisions are customarily a result of the Reviewing Editor and the peer reviewers coming together and discussing their recommendations until a consensus is reached. When revisions are invited, a fact-based synthesis statement explaining their decision and outlining what is needed to prepare a revision will be listed below. The following reviewer(s) agreed to reveal their identity: Brandon Henderson.

This manuscript has been reviewed by three experts in the field. All reviewers agreed that important findings were reported in the manuscript. The studies focus on VTA dopaminergic neurons that project to the interpenducular nucleus (IPN), a circuit whose presence has been questioned. This manuscript provides evidence for how dopamine release in this region is related to behavior. It was noted that most of the general results have been published before, but they were called into question by another paper. Thus, the current paper is a confirmatory study, rather than a novel investigation that advances the field. In the consultation session, concerns were raised about the research methods, image saturation and lack of quantification, and data inclusion/exclusion criteria. It was further agreed that the custom-written MatLab scripts should be made available for validation of data processing. A statistics table is also required per eNeuro guidelines and reviewer requests. Detailed reviewer concerns are as follows:

Reviewer 1: This manuscript focuses on VTA dopaminergic neurons that project to the interpenducular nucleus (IPN), a circuit whose existence has been questioned, and which has been shown previously to have roles in anxiety-like behavior and novelty preference. The authors record immunohistochemical approaches with fiber-based recording techniques to characterize this circuit and recorded dopamine via a genetically encoded dopamine sensor in a social investigation task. I am excited about the investigation of the role of dopamine outside of the canonical brain regions that are typically studied, as well as what the authors think that dopamine is doing in this area. Weaknesses hinge around limited presentation of the data which makes it difficult to determine what is going on (specifics below). Further, the manuscript warrants further discussion about how these data fit into the idea of saliency and broader contexts for the field. Overall, I am excited about the topic and approaches used in the manuscript, however, it is not without weakness. I hope the authors view my comments as constructive and in good faith as they are intended. I am not negative about the questions or even the data, however, I do want to ensure that the authors have thought of the potential confounding variables in their analysis and want to take the appropriate measures to rule them out. I have listed my main points as major and minor below:

Major

1) There are major conclusions made using a series of immunohistochemistry figures. I have several concerns:

a) The GFP images are very oversaturated, I cannot tell if this happened in the image acquisition or processing stage, but it makes it very difficult to evaluate.

b) No quantification is presented of these images, even though most of the conclusions of the manuscript are based on these data.

c) The authors present several animals, but not all of the ones that were imaged. It is unclear how the animals that are presented were selected.

2) The largest concerns are around the fiber photometry data. This is a very small projection and even in the images presented in earlier figures make it is clear that there are only single axons in some fields. Using fiber photometry, the signal may be too low to get an accurate assessment of task-relevant fluctuations in dopamine levels that rise above the other factors that interact with these approaches when there is a lack of spatial resolution. However, because there is not sufficient information to understand exactly what was done it is difficult to give very specific feedback to guide the authors. Below I have listed some issues and things that they need to address to ensure that the data are valid.

a) The z-scores are very small. If you don’t have significant task-relevant events in individual groups, how can make conclusions about differences across groups and map them onto latent variables like salience? I understand the data are the data; however, I want to be sure that these data represent what the authors are concluding and not an artifact of averaging multiple trials, bloodflow, or other confounding factors that need to be ruled out to ensure the veracity of the data.

b) The biggest concern here is the trial analysis that was done in an unstructured task. There is a large possibility, based on the window for analysis that the authors chose. that the authors are averaging the same data over and over by accident. For example, if you were imaging during licks in a sucrose task, and you average the signal around licks you will introduce the same data as independent replicates into the analysis as mice lick in bouts. A single bout may have 200 licks (but it is variable), so you need to make sure that you don’t just align around each lick and then average 200 datapoints that all have overlapping data traces in them. This artificially increases your power and makes the data difficult to interpret. I am worried that that is happening here since social interaction is similarly variable and has bouts of behavior that differ in length. I think an interesting analysis that would both address this and is theoretically interesting is to pick the first interaction. Or do a comparison between the first and the last. Do you get an effect only on the first and does it go down significantly over repeated interactions.

c) Why are there the exact same number of social events to the familiar animal?

d) Task variables have been shown to increase cerebral blood flow which can alter the signal in a way that is not dependent on the biosensor through reflection (10.1016/j.crmeth.2022.100243). While in some brain regions and cell populations the signals are very large and likely overshadow these effects, here the neural signal that is being seen is very small. I would suggest the authors present unprocessed data - just the 470 and 405 channels alone at the very least and think of potential ways to show that this is the signal that they thing it is.

e) At the very least more unprocessed data needs to shown to convince the reader that these are real and behaviorally relevant events that are because dopamine is being release. Some things that should be done to show this: 1. Random sampling of the same window over the data (so take the same length of time at random). If the effect you are seeing is task-relevant you should see a difference between the random sampling and the task events. 2) average the isosbestic channel in the same way that you did for the analyzed data around the same timepoints to show no effect.

3) There is not sufficient evidence provided within this manuscript to suggest that dopamine release encodes perceived salience in the IPN. This is not to say that it doesn’t. But a discussion in the paper should be made about why they think this. What is being studied in this paper are state variables (anxiety etc). These included temporal processing variables like salience - so this could be salience - but the data in the paper does not prove that. That is fine. But in the discussion the authors should synthesize work from field to better explain why they think this is what the IPN does.

Minor

4) Are the binned analysis of z-scores appropriate for statistical tests in figures 4 and 5?

Reviewer 2:

The authors describe a novel pathway that projects from the VTA to the IPN and this appears to be specific to dopamine projections. As presented, this would be a significant increase in our knowledge and our understanding of the habenular-IPN-VTA circuit and its relation to motivated behaviors. However, there are some issues that need to be resolved to strengthen this work:

Major Comments:

1. The authors state that for the first validation experiment, “the 4 most relevant cases are presented.” However, they do not provide justification for why cases 5 and 6 were not presented. Given that there is no size-restriction imposed on authors, all of the ‘cases’ should be presented in some fashion or the authors need to provide a clear justification for why they are not presented.

2. Similarly, for the second validation experiment, “the 3 most relevant cases are presented.” Here, they exclude 3 cases without any further discussion. Just as stated above, these cases need to be included or a strong justification for not presenting them needs to be provided.

For the two above comments, if the authors simply want to exclude them to prevent their figures from being too large, the other cases could be presented as extended data if that falls under the guidelines of eNeuro.

3. Unless I am mistaken, it is an eNeuro policy to have a complete table of statistics included with the manuscript.

4. The authors come to the conclusion that this pathway can be implicated in salience; but I believe that additional experiments need to be included to fully implicate this circuit in behaviors related to salience. It is clear that this circuit may impact novelty and anxiety-related behaviors; but implicating salience may be stretching the observations of the current study.

Minor Comments:

1. The authors mention that male and female mice were used; however, there is no clear description of their numbers. In the figure legends, it merely states something like ‘n = 10 - 11 mice’. The authors should clearly describe how many male and female mice were used for each experiment in the legends.

2. (very minor comment) For stereotaxic surgeries, was a heat pad used following the use of ketamine/xylazine. If so, this should be included in the methods.

3. Methods, immunostaining and microscopy - The authors state they used a confocal microscope “at 10X or 1.5 zoom”. I believe the authors should amend this to “at 10X or 10X with a 1.5 zoom” to clarify that the 1.5 zoom was applied to a 10X objective.

Reviewer 3:

This is an authorship-redacted study with the goal of confirming previous findings that show that VTA neurons can project to the local/adjacent IPN. This issue was recently called into question by another paper (Nasirova et al). In the present study, the investigators used DAT-Cre mice and viral techniques to show or confirm that VTA DA neurons in mice send sparse projections to the caudal/dorsal IPN. They then use dLight imaging to show that there are measurable dLight signals in IPN during several behavioral tasks that are known to elicit DA neuron activation. There are strengths and weaknesses, the latter noted below. The study will have some value in confirming and refining the idea that VTA neurons project to the IPN. This is relevant, as this idea was directly challenged by Nasirova et al. However, this paper does not extend our understanding of the functional relevance of a VTA to IPN projection, assuming it exists as the paper asserts. Regardless, the paper is valuable as a confirmation study.

Points for consideration:

Figure 1

-axonal targeted construct to show presynaptic expression in IPN

-4 cases only for VTA-DA to IPN in Figure 1...no attempt at quantification or comparison to a control, lowering the quality of the evidence (basically a case study)...what makes a case “relevant” or not? Whether it showed the desired result? The paper should do a better job of explaining the criteria for inclusion or exclusion of cases as “relevant”.

-IPN innervation is not extensive, suggesting the question: how important is it? This is mitigated by consideration of cholinergic interneurons in striatum, which are sparse yet important. However, we don’t understand the importance of the VTA to IPN projection like we understand cholinergic interneurons.

Figure 2

-repeated Figure 1 experiment with axon-targeted GCaMP, resulting in weak labeling of GCaMP-positive structures in IPN that co-label with synaptophysin protein (images appear overexposed, detracting from the quality of the evidence a bit)

-only single image shown, no quantification. Figure 1 and 2 would be strengthened by inclusion of quantification rather than single images of specific cases whose criteria for inclusion/exclusion is unclear.

Figure 3

-retro GFP injected into NAc to back-label VTA-DA, then look at whether there were forward axonal projections in IPN...

-“relevant” cases presented (what is relevant vs. irrelevant?)

Figure 4/5

-dLight measurements crucially depends on excluding any contaminating signal from the VTA, as there could be somatic signal from the VTA. This is especially relevant given the recent paper (Nasirova et al) that directly contradicts the main claim of this paper. The paper states that all animals were verified, but no data was presented to substantiate this assertion.

-Assuming the data in the paper are accurate (see above for reasons to be less than 100% confident), the paper would be stronger if it showed that the VTA to IPN projection was important for some biological process. This was shown, however, in a previous paper. This paper appears to have the goal of rebutting Nasirova et al, which questioned the result that VTA neurons send projections to IPN.

## References

[B1] Ables JL, Görlich A, Antolin-Fontes B, Wang C, Lipford SM, Riad MH, Ren J, Hu F, Luo M, Kenny PJ, Heintz N, Ibañez-Tallon I (2017) Retrograde inhibition by a specific subset of interpeduncular α5 nicotinic neurons regulates nicotine preference. Proc Natl Acad Sci U S A 114:13012–13017. 10.1073/pnas.1717506114 29158387 PMC5724287

[B2] Adell A, Artigas F (2004) The somatodendritic release of dopamine in the ventral tegmental area and its regulation by afferent transmitter systems. Neurosci Biobehav Rev 28:415–431. 10.1016/j.neubiorev.2004.05.001 15289006

[B3] Antolin-Fontes B, Ables JL, Görlich A, Ibañez-Tallon I (2015) The habenulo-interpeduncular pathway in nicotine aversion and withdrawal. Neuropharmacology 96:213–222. 10.1016/j.neuropharm.2014.11.019 25476971 PMC4452453

[B4] Aransay A, Rodríguez-López C, García-Amado M, Clascá F, Prensa L (2015) Long-range projection neurons of the mouse ventral tegmental area: a single-cell axon tracing analysis. Front Neuroanat 9:59. 10.3389/fnana.2015.00059 26042000 PMC4436899

[B5] Arber S, Costa RM (2022) Networking brainstem and basal ganglia circuits for movement. Nat Rev Neurosci 23:342–360. 10.1038/s41583-022-00581-w 35422525

[B6] Bäckman CM, Malik N, Zhang YJ, Shan L, Grinberg A, Hoffer BJ, Westphal H, Tomac AC (2006) Characterization of a mouse strain expressing Cre recombinase from the 3′ untranslated region of the dopamine transporter locus. Genesis 44:383–390. 10.1002/dvg.20228 16865686

[B7] Bariselli S, Hörnberg H, Prévost-Solié C, Musardo S, Hatstatt-Burklé L, Scheiffele P, Bellone C (2018) Role of VTA dopamine neurons and neuroligin 3 in sociability traits related to nonfamiliar conspecific interaction. Nat Commun 9:3173 10.1038/s41467-018-05382-3 30093665 PMC6085391

[B8] Beier KT, Steinberg EE, Deloach KE, Xie S, Miyamichi K, Schwarz L, Gao XJ, Kremer EJ, Malenka RC, Luo L (2015) Circuit architecture of VTA dopamine neurons revealed by systematic input-output mapping. Cell 162:622–634. 10.1016/j.cell.2015.07.015 26232228 PMC4522312

[B9] Berke JD (2018) What does dopamine mean? Nat Neurosci 21:787–793. 10.1038/s41593-018-0152-y 29760524 PMC6358212

[B10] Berrettini W, Yuan X, Tozzi F, Song K, Francks C, Chilcoat H, Waterworth D, Muglia P, Mooser V (2008) α-5/α-3 nicotinic receptor subunit alleles increase risk for heavy smoking. Mol Psychiatry 13:368–373. 10.1038/sj.mp.4002154 18227835 PMC2507863

[B11] Berridge KC, Robinson TE (1998) What is the role of dopamine in reward: hedonic impact, reward learning, or incentive salience? Brain Res Rev 28:309–369. 10.1016/S0165-0173(98)00019-8 9858756

[B12] Bierut LJ, et al. (2008) Variants in nicotinic receptors and risk for nicotine dependence. Am J Psychiatry 165:1163–1171. 10.1176/appi.ajp.2008.07111711 18519524 PMC2574742

[B13] Broussard GJ, Liang Y, Fridman M, Unger EK, Meng G, Xiao X, Ji N, Petreanu L, Tian L (2018) In vivo measurement of afferent activity with axon-specific calcium imaging. Nat Neurosci 21:1272–1280. 10.1038/s41593-018-0211-4 30127424 PMC6697169

[B14] Casserly AP, Tsuji J, Zhao-Shea R, Smith CB, Molas S, Tapper AR, Weng Z, Gardner PD (2020) Integrated miRNA-/mRNA-seq of the habenulo-interpeduncular circuit during acute nicotine withdrawal. Sci Rep 10:813. 10.1038/s41598-020-57907-w 31965003 PMC6972841

[B15] Chevallier C, Kohls G, Troiani V, Brodkin ES, Schultz RT (2012) The social motivation theory of autism. Trends Cogn Sci 16:231–239. 10.1016/j.tics.2012.02.007 22425667 PMC3329932

[B16] Dalgleish HWP, Russell LE, Packer AM, Roth A, Gauld OM, Greenstreet F, Thompson EJ, Häusser M (2020) How many neurons are sufficient for perception of cortical activity? Elife 9:e58889. 10.7554/eLife.58889 33103656 PMC7695456

[B17] de Jong JW, Afjei SA, Pollak Dorocic I, Peck JR, Liu C, Kim CK, Tian L, Deisseroth K, Lammel S (2019) A neural circuit mechanism for encoding aversive stimuli in the mesolimbic dopamine system. Neuron 101:133–151.e7. 10.1016/j.neuron.2018.11.005 30503173 PMC6317997

[B18] DeGroot SR, Zhao-Shea R, Chung L, Klenowski PM, Sun F, Molas S, Gardner PD, Li Y, Tapper AR (2020) Midbrain dopamine controls anxiety-like behavior by engaging unique interpeduncular nucleus microcircuitry. Biol Psychiatry 88:855–866. 10.1016/j.biopsych.2020.06.018 32800629 PMC8043246

[B19] Ferreira JGP, Del-Fava F, Hasue RH, Shammah-Lagnado SJ (2008) Organization of ventral tegmental area projections to the ventral tegmental area–nigral complex in the rat. Neuroscience 153:196–213. 10.1016/j.neuroscience.2008.02.003 18358616

[B20] Flagel SB, Clark JJ, Robinson TE, Mayo L, Czuj A, Willuhn I, Akers CA, Clinton SM, Phillips PEM, Akil H (2011) A selective role for dopamine in stimulus–reward learning. Nature 469:53–57. 10.1038/nature09588 21150898 PMC3058375

[B21] Floresco SB (2015) The nucleus accumbens: an interface between cognition, emotion, and action. Annu Rev Psychol 66:25–52. 10.1146/annurev-psych-010213-115159 25251489

[B22] Fowler CD, Lu Q, Johnson PM, Marks MJ, Kenny PJ (2011) Habenular α5 nicotinic receptor subunit signalling controls nicotine intake. Nature 471:597–601. 10.1038/nature09797 21278726 PMC3079537

[B23] Frahm S, Ślimak MA, Ferrarese L, Santos-Torres J, Antolin-Fontes B, Auer S, Filkin S, Pons S, Fontaine JF, Tsetlin V, Maskos U, Ibañez-Tallon I (2011) Aversion to nicotine is regulated by the balanced activity of β4 and α5 nicotinic receptor subunits in the medial habenula. Neuron 70:522–535. 10.1016/j.neuron.2011.04.013 21555077

[B24] Görlich A, Antolin-Fontes B, Ables JL, Frahm S, Ślimak MA, Dougherty JD, Ibañez-Tallon I (2013) Reexposure to nicotine during withdrawal increases the pacemaking activity of cholinergic habenular neurons. Proc Natl Acad Sci U S A 110:17077–17082. 10.1073/pnas.1313103110 24082085 PMC3800986

[B25] Groenewegen HJ, Ahlenius S, Haber SN, Kowall NW, Nauta WJH (1986) Cytoarchitecture, fiber connections, and some histochemical aspects of the interpeduncular nucleus in the rat. J Comp Neurol 249:65–102. 10.1002/cne.902490107 2426312

[B26] Gunaydin LA, Grosenick L, Finkelstein JC, Kauvar I. v, Fenno LE, Adhikari A, Lammel S, Mirzabekov JJ, Airan RD, Zalocusky KA, Tye KM, Anikeeva P, Malenka RC, Deisseroth K (2014) Natural neural projection dynamics underlying social behavior. Cell 157:1535–1551. 10.1016/j.cell.2014.05.017 24949967 PMC4123133

[B27] Hemmendinger LM, Moore RY (1984) Interpeduncular nucleus organization in the rat: cytoarchitecture and histochemical analysis. Brain Res Bull 13:163–179. 10.1016/0361-9230(84)90018-2 6148133

[B28] Heymann G, Jo YS, Reichard KL, McFarland N, Chavkin C, Palmiter RD, Soden ME, Zweifel LS (2020) Synergy of distinct dopamine projection populations in behavioral reinforcement. Neuron 105:909–920.e5. 10.1016/j.neuron.2019.11.024 31879163 PMC7060117

[B29] Horga G, Abi-Dargham A (2019) An integrative framework for perceptual disturbances in psychosis. Nat Rev Neurosci 20:763–778. 10.1038/s41583-019-0234-1 31712782

[B30] Hung LW, Neuner S, Polepalli JS, Beier KT, Wright M, Walsh JJ, Lewis EM, Luo L, Deisseroth K, Dölen G, Malenka RC (2017) Gating of social reward by oxytocin in the ventral tegmental area. Science 357:1406–1411. 10.1126/science.aan4994 28963257 PMC6214365

[B31] Improgo MRD, Scofield MD, Tapper AR, Gardner PD (2010) The nicotinic acetylcholine receptor CHRNA5/A3/B4 gene cluster: dual role in nicotine addiction and lung cancer. Prog Neurobiol 92:212–226. 10.1016/j.pneurobio.2010.05.003 20685379 PMC2939268

[B32] Klenowski PM, Zhao-Shea R, Freels TG, Molas S, Tapper AR (2022) Dynamic activity of interpeduncular nucleus GABAergic neurons controls expression of nicotine withdrawal in male mice. Neuropsychopharmacology 47:641–651. 10.1038/s41386-021-01107-1 34326477 PMC8782840

[B33] Kutlu MG, Zachry JE, Melugin PR, Cajigas SA, Chevee MF, Kelly SJ, Kutlu B, Tian L, Siciliano CA, Calipari ES (2021) Dopamine release in the nucleus accumbens core signals perceived saliency. Curr Biol 31:4748–4761.e8. 10.1016/j.cub.2021.08.052 34529938 PMC9084920

[B34] Lammel S, Hetzel A, Häckel O, Jones I, Liss B, Roeper J (2008) Unique properties of mesoprefrontal neurons within a dual mesocorticolimbic dopamine system. Neuron 57:760–773. 10.1016/j.neuron.2008.01.022 18341995

[B35] Lammel S, Ion DI, Roeper J, Malenka RC (2011) Projection-specific modulation of dopamine neuron synapses by aversive and rewarding stimuli. Neuron 70:855–862. 10.1016/j.neuron.2011.03.025 21658580 PMC3112473

[B36] Lammel S, Lim BK, Ran C, Huang KW, Betley MJ, Tye KM, Deisseroth K, Malenka RC (2012) Input-specific control of reward and aversion in the ventral tegmental area. Nature 491:212–217. 10.1038/nature11527 23064228 PMC3493743

[B37] Lammel S, Lim BK, Malenka RC (2014) Reward and aversion in a heterogeneous midbrain dopamine system. Neuropharmacology 76:351–359. 10.1016/j.neuropharm.2013.03.019 23578393 PMC3778102

[B38] Lammel S, Steinberg EE, Földy C, Wall NR, Beier K, Luo L, Malenka RC (2015) Diversity of transgenic mouse models for selective targeting of midbrain dopamine neurons. Neuron 85:429–438. 10.1016/j.neuron.2014.12.036 25611513 PMC5037114

[B39] Lima LB, Bueno D, Leite F, Souza S, Gonçalves L, Furigo IC, Donato J, Metzger M (2017) Afferent and efferent connections of the interpeduncular nucleus with special reference to circuits involving the habenula and raphe nuclei. J Comp Neurol 525:2411–2442. 10.1002/cne.24217 28340505

[B40] Marshel JH, Kim YS, Machado TA, Quirin S, Benson B, Kadmon J, Raja C, Chibukhchyan A, Ramakrishnan C, Inoue M, Shane JC, McKnight DJ, Yoshizawa S, Kato HE, Ganguli S, Deisseroth K (2019) Cortical layer-specific critical dynamics triggering perception. Science 365:eaaw52002. 10.1126/science.aaw5202 PMC6711485 31320556

[B41] Matsumoto M, Hikosaka O (2009) Two types of dopamine neuron distinctly convey positive and negative motivational signals. Nature 459:837–841. 10.1038/nature08028 19448610 PMC2739096

[B42] Mirenowicz J, Schultz W (1996) Preferential activation of midbrain dopamine neurons by appetitive rather than aversive stimuli. Nature 379:449–451. 10.1038/379449a0 8559249

[B43] Molas S, DeGroot SR, Zhao-Shea R, Tapper AR (2017a) Anxiety and nicotine dependence: emerging role of the habenulo-interpeduncular axis. Trends Pharmacol Sci 38:169–180. 10.1016/j.tips.2016.11.001 27890353 PMC5258775

[B44] Molas S, Zhao-Shea R, Liu L, Degroot SR, Gardner PD, Tapper AR (2017b) A circuit-based mechanism underlying familiarity signaling and the preference for novelty. Nat Neurosci 20:1260–1268. 10.1038/nn.4607 28714952 PMC5752132

[B45] Morales M, Margolis EB (2017) Ventral tegmental area: cellular heterogeneity, connectivity and behaviour. Nat Rev Neurosci 18:73–85. 10.1038/nrn.2016.165 28053327

[B46] Nasirova N, Quina LA, Novik S, Turner EE (2021) Genetically targeted connectivity tracing excludes dopaminergic inputs to the interpeduncular nucleus from the ventral tegmentum and substantia nigra. eNeuro 8:ENEURO.0127-21.2021. 10.1523/ENEURO.0127-21.2021 PMC8223495 34088738

[B47] Nestler EJ, Lüscher C (2019) The molecular basis of drug addiction: linking epigenetic to synaptic and circuit mechanisms. Neuron 102:48–59. 10.1016/j.neuron.2019.01.016 30946825 PMC6587180

[B48] Okuyama T, Kitamura T, Roy DS, Itohara S, Tonegawa S (2016) Ventral CA1 neurons store social memory. Science 353:1536–1541. 10.1126/science.aaf7003 27708103 PMC5493325

[B49] Patriarchi T, Cho JR, Merten K, Howe MW, Marley A, Xiong WH, Folk RW, Broussard GJ, Liang R, Jang MJ, Zhong H, Dombeck D, von Zastrow M, Nimmerjahn A, Gradinaru V, Williams JT, Tian L (2018) Ultrafast neuronal imaging of dopamine dynamics with designed genetically encoded sensors. Science 360:eaat4422. 10.1126/science.aat4422 29853555 PMC6287765

[B50] Paxinos G, Franklin KBJ (2001) The mouse brain in stereotaxic coordinates, Ed 2. San Diego: Academic.

[B51] Phillips RA, Tuscher JJ, Black SL, Andraka E, Fitzgerald ND, Ianov L, Day JJ (2022) An atlas of transcriptionally defined cell populations in the rat ventral tegmental area. Cell Rep 39:110616. 10.1016/j.celrep.2022.110616 35385745 PMC10888206

[B52] Phillipson OT, Pycock CJ (1982) Dopamine neurones of the ventral tegmentum project to both medial and lateral habenula. Exp Brain Res 45:89–94. 6799315 10.1007/BF00235766

[B53] Poulin JF, Caronia G, Hofer C, Cui Q, Helm B, Ramakrishnan C, Chan CS, Dombeck DA, Deisseroth K, Awatramani R (2018) Mapping projections of molecularly defined dopamine neuron subtypes using intersectional genetic approaches. Nat Neurosci 21:1260–1271. 10.1038/s41593-018-0203-4 30104732 PMC6342021

[B54] Poulin JF, Gaertner Z, Moreno-Ramos OA, Awatramani R (2020) Classification of midbrain dopamine neurons using single-cell gene expression profiling approaches. Trends Neurosci 43:155–169. 10.1016/j.tins.2020.01.004 32101709 PMC7285906

[B55] Powell JM, Plummer NM, Scappini EL, Tucker CJ, Jensen P (2019) DEFiNE: a method for enhancement and quantification of fluorescently labeled axons. Front Neuroanat 12:117. 30687025 10.3389/fnana.2018.00117 PMC6336715

[B56] Quina LA, Harris J, Zeng H, Turner EE (2017) Specific connections of the interpeduncular subnuclei reveal distinct components of the habenulopeduncular pathway. J Comp Neurol 525:2632–2656. 10.1002/cne.24221 28387937 PMC5873981

[B57] Ruitenberg MFL, van Wouwe NC, Wylie SA, Abrahamse EL (2021) The role of dopamine in action control: insights from medication effects in Parkinson’s disease. Neurosci Biobehav Rev 127:158–170. 10.1016/j.neubiorev.2021.04.023 33905788

[B58] Salas R, Sturm R, Boulter J, de Biasi M (2009) Nicotinic receptors in the habenulo-interpeduncular system are necessary for nicotine withdrawal in mice. J Neurosci 29:3014–3018. 10.1523/JNEUROSCI.4934-08.2009 19279237 PMC3862238

[B59] Saunders BT, Richard JM, Margolis EB, Janak PH (2018) Dopamine neurons create Pavlovian conditioned stimuli with circuit-defined motivational properties. Nat Neurosci 21:1072–1083. 10.1038/s41593-018-0191-4 30038277 PMC6082399

[B60] Schultz W, Dayan P, Montague PR (1997) A neural substrate of prediction and reward. Science 275:1593–1599. 10.1126/science.275.5306.1593 9054347

[B61] Seigneur E, Polepalli JS, Südhof TC (2018) Cbln2 and Cbln4 are expressed in distinct medial habenula-interpeduncular projections and contribute to different behavioral outputs. Proc Natl Acad Sci U S A 115:E10235–E10244. 10.1073/pnas.1811086115 30287486 PMC6205418

[B62] Sherafat Y, Bautista M, Fowler JP, Chen E, Ahmed A, Fowler CD (2020) The interpeduncular-ventral hippocampus pathway mediates active stress coping and natural reward. eNeuro 7:ENEURO.0191-20.2020–17. 10.1523/ENEURO.0191-20.2020 PMC7688303 33139320

[B63] Solié C, Girard B, Righetti B, Tapparel M, Bellone C (2022) VTA dopamine neuron activity encodes social interaction and promotes reinforcement learning through social prediction error. Nat Neurosci 25:86–97. 10.1038/s41593-021-00972-9 34857949 PMC7612196

[B64] Soria-Gómez E, Busquets-Garcia A, Hu F, Mehidi A, Cannich A, Roux L, Louit I, Alonso L, Wiesner T, Georges F, Verrier D, Vincent P, Ferreira G, Luo M, Marsicano G (2015) Habenular CB1 receptors control the expression of aversive memories. Neuron 88:306–313. 10.1016/j.neuron.2015.08.035 26412490

[B65] Swanson LW (1982) The projections of the ventral tegmental area and adjacent regions: a combined fluorescent retrograde tracer and immunofluorescence study in the rat. Brain Res Bull 9:321–353. 10.1016/0361-9230(82)90145-9 6816390

[B66] Taylor WD, Zald DH, Felger JC, Christman S, Claassen DO, Horga G, Miller JM, Gifford K, Rogers B, Szymkowicz SM, Rutherford BR (2022) Influences of dopaminergic system dysfunction on late-life depression. Mol Psychiatry 27:180–191. 10.1038/s41380-021-01265-0 34404915 PMC8850529

[B67] Tiklová K, Björklund ÅK, Lahti L, Fiorenzano A, Nolbrant S, Gillberg L, Volakakis N, Yokota C, Hilscher MM, Hauling T, Holmström F, Joodmardi E, Nilsson M, Parmar M, Perlmann T (2019) Single-cell RNA sequencing reveals midbrain dopamine neuron diversity emerging during mouse brain development. Nat Commun 10:581. 10.1038/s41467-019-08453-1 30718509 PMC6362095

[B68] Walf AA, Frye CA (2007) The use of the elevated plus maze as an assay of anxiety-related behavior in rodents. Nat Protoc 2:322–328. 10.1038/nprot.2007.44 17406592 PMC3623971

[B69] Watabe-Uchida M, Zhu L, Ogawa SK, Vamanrao A, Uchida N (2012) Whole-brain mapping of direct inputs to midbrain dopamine neurons. Neuron 74:858–873. 10.1016/j.neuron.2012.03.017 22681690

[B70] Wills L, Ables JL, Braunscheidel KM, Caligiuri SPB, Elayouby KS, Fillinger C, Ishikawa M, Moen JK, Kenny PJ (2022) Neurobiological mechanisms of nicotine reward and aversion. 74:271–310. 10.1124/pharmrev.121.000299 35017179 PMC11060337

[B71] Yamaguchi T, Danjo T, Pastan I, Hikida T, Nakanishi S (2013) Distinct roles of segregated transmission of the septo-habenular pathway in anxiety and fear. Neuron 78:537–544. 10.1016/j.neuron.2013.02.035 23602500 PMC3654012

[B72] Zalachoras I, Astori S, Meijer M, Grosse J, Zanoletti O, de Suduiraut IG, Deussing JM, Sandi C (2022) Opposite effects of stress on effortful motivation in high and low anxiety are mediated by CRHR1 in the VTA. Sci Adv 8:abj9019. 10.1126/sciadv.abj9019 PMC8942367 35319997

[B73] Zhang J, Tan L, Ren Y, Liang J, Lin R, Feng Q, Zhou J, Hu F, Ren J, Wei C, Yu T, Zhuang Y, Bettler B, Wang F, Luo M (2016a) Presynaptic excitation via GABA_B_ receptors in habenula cholinergic neurons regulates fear memory expression. Cell 166:716–728. 10.1016/j.cell.2016.06.026 27426949

[B74] Zhang S, Xu M, Chang WC, Ma C, Hoang Do JP, Jeong D, Lei T, Fan JL, Dan Y (2016b) Organization of long-range inputs and outputs of frontal cortex for top-down control. Nat Neurosci 19:1733–1742. 10.1038/nn.4417 27749828 PMC5127741

[B75] Zhao-Shea R, Liu L, Pang X, Gardner PD, Tapper AR (2013) Activation of GABAergic neurons in the interpeduncular nucleus triggers physical nicotine withdrawal symptoms. Curr Biol 23:2327–2335. 10.1016/j.cub.2013.09.041 24239118 PMC3855889

[B76] Zhao-Shea R, Degroot SR, Liu L, Vallaster M, Pang X, Su Q, Gao G, Rando OJ, Martin GE, George O, Gardner PD, Tapper AR (2015) Increased CRF signalling in a ventral tegmental area-interpeduncular nucleus-medial habenula circuit induces anxiety during nicotine withdrawal. Nat Commun 6:6770. 10.1038/ncomms7770 25898242 PMC4405813

[B77] Zweifel LS, Fadok JP, Argilli E, Garelick MG, Jones GL, Dickerson TMK, Allen JM, Mizumori SJY, Bonci A, Palmiter RD (2011) Activation of dopamine neurons is critical for aversive conditioning and prevention of generalized anxiety. Nat Neurosci 14:620–626. 10.1038/nn.2808 21499253 PMC3083461

